# Thermal stress jeopardizes carbonate production of coral reefs across the western and central Pacific Ocean

**DOI:** 10.1371/journal.pone.0249008

**Published:** 2021-04-26

**Authors:** Robert van Woesik, Christopher William Cacciapaglia

**Affiliations:** Institute for Global Ecology, Florida Institute of Technology, Melbourne, Florida, United States of America; Newcastle University, UNITED KINGDOM

## Abstract

Coral reefs protect islands, coastal areas, and their inhabitants from storm waves and provide essential goods and services to millions of people worldwide. Yet contemporary rates of ocean warming and local disturbances are jeopardizing the reef-building capacity of coral reefs to keep up with rapid rates of sea-level rise. This study compared the reef-building capacity of shallow-water habitats at 142 sites across a potential thermal-stress gradient in the tropical Pacific Ocean. We sought to determine the extent to which habitat differences and environmental variables potentially affect rates of net carbonate production. In general, outer-exposed reefs and lagoonal-patch reefs had higher rates of net carbonate production than nearshore reefs. The study found that thermal anomalies, particularly the intensity of thermal-stress events, play a significant role in reducing net carbonate production—evident as a diminishing trend of net carbonate production from the western to the central tropical Pacific Ocean. The results also showed a latent spatial effect along the same gradient, not explained by thermal stress, suggesting that reefs in the western tropical Pacific Ocean are potentially enhanced by the proximity of reefs in the Coral Triangle—an effect that diminishes with increasing distance and isolation.

## Introduction

Coral reefs reduce storm-driven wave energy by over 95% [1] protecting island and coastal inhabitants and provide other essential goods and services to millions of people worldwide [2]. Yet, after more than 5000 years of relative sea-level stability [3, 4], coral reefs worldwide are currently experiencing rapid rates of sea-level rise [5, 6]. In the geological past, healthy reefs tracked sea-level fluctuations [7], but recent increases in thermal-stress events [8, 9] and local disturbances [10] are reducing the capacity of coral reefs to keep up with sea-level rise [11, 12]. Therefore, one of the central questions today is: where will coral reefs be able to accumulate enough carbonate to keep up with the rate of sea-level rise? This question is particularly relevant as the average rate of sea-level rise is expected to increase from 2 mm to 9 mm a year into the 21^st^ century [13–15].

Historically, coral reefs have grown where the rates of incremental buildup of calcium carbonate, deposited by calcifying organisms such as corals and coralline algae, exceed the rates of physical, chemical, and biological erosion [16–22]. Over the last 10,000 years, through the Holocene, modal rates of net carbonate production of Indo-Pacific reefs have been estimated at ~ 10 kg CaCO_3_ m^-2^ y^-1^, which equates to ~ 7 mm y^-1^ of lateral expansion [23–26]. Yet there are regional and local differences in rates of carbonate production [25, 27]. For example, in the modern Caribbean, average net carbonate production rates (~1.5 kg CaCO_3_ m^-2^ y^-1^ [11]) are considerably lower than average carbonate production rates in the western Pacific Ocean (~ 9.7 kg CaCO_3_ m^-2^ y^-1^, [28]). There are also major differences in net carbonate production across reef habitats [27]. For example, the outer reefs of Palau and Yap, in the western Pacific, have higher average rates of net carbonate production of ~10 CaCO_3_ kg m^-2^ y^-1^ than the inner reefs (i.e., nearshore reefs) of the same islands at ~ 7 CaCO_3_ kg m^-2^ y^-1^ [28]. Carbonate production is also dependent on a suite of other interacting variables, including depth, macroalgal presence, and the abundance of excavating parrotfish [29].

As in the past, current and future rates of reef growth will depend on the persistence and the density of reef-building corals [7, 24, 30]. The most significant influence that reduces the density of reef-building corals are thermal-stress events [29]. Thermal-stress events are causing coral bleaching and mortality that are changing reef composition [31–35] and are reducing the capacity of reefs to grow and keep up with sea-level rise [29].

To date, most studies that have recorded spatial differences in carbonate production have been localized [28, 29, 36, 37], or focused on the Caribbean [11]. No studies have assessed carbonate production across large expanses of the Pacific Ocean. Here we use a field-based approach to examine carbonate production at 142 coral-reef sites spanning across the western and central tropical Pacific Ocean. We examined whether thermal stress alone is limiting carbonate production, or whether local and regional differences in carbonate production can be attributed to other environmental factors (including habitat); and whether there are any latent spatial effects not explained by thermal stress.

## Methods

### Field methods

**Fig 1:**
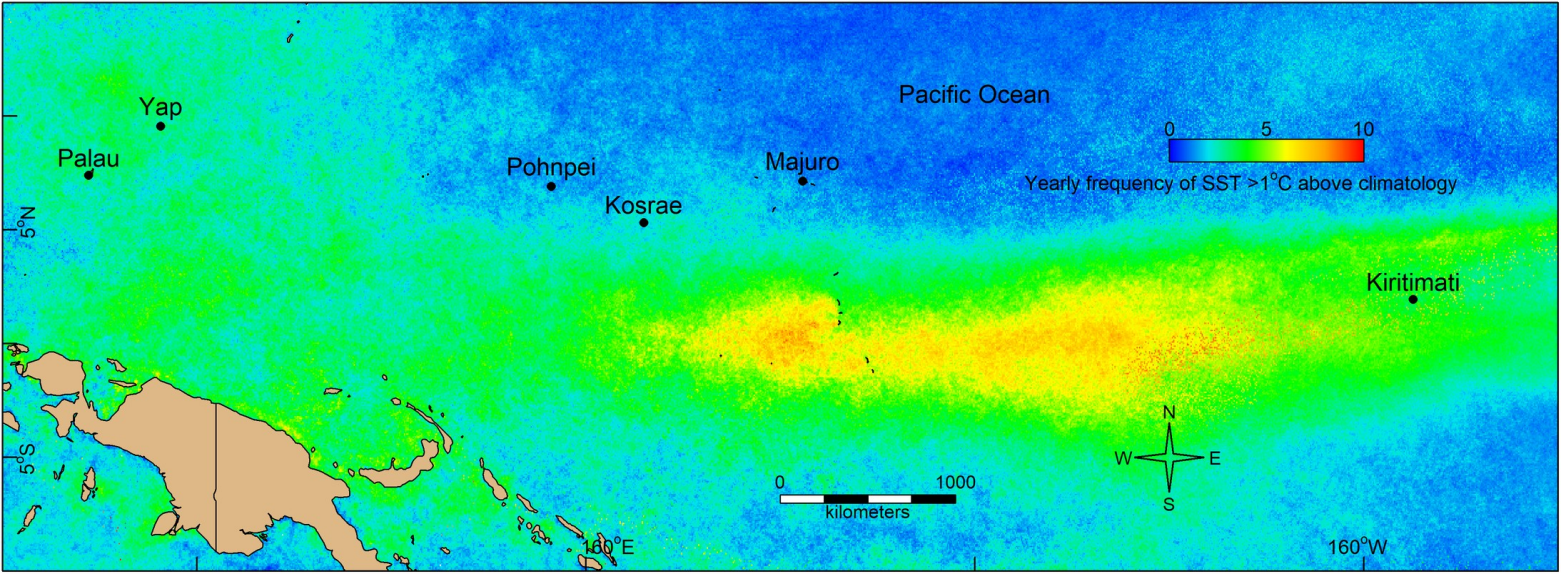
The six Pacific Ocean study locations at Palau, Yap, Pohnpei, Kosrae, Majuro and Kiritimati. The background image captures the frequency per year that the weekly sea-surface temperatures (SSTs) (from CoRTAD version 6, [39]) were greater than 1°C above the relative climatology, averaged from 6 January 2000 to 26 December 2019. Outline of land plotted from the R package ‘maptools’ [63].

Underwater surveys were conducted on the reefs of Palau (Republic of Palau) and Yap in 2017, Pohnpei and Kosrae (Federated States of Micronesia) in 2018, and Majuro (Republic of the Marshall Islands) and Kiritimati (Republic of Kiribati) in 2019 (Fig 1). In all locations, a stratified random sampling approach was used to survey the reefs for carbonate production by randomly selecting 24 sites on each island, with the exception of Kiritimati where only 22 of the 24 sites were surveyed because of inclement weather. At all locations, we stratified the sites as either (i) outer reefs, (ii) patch reefs in lagoons, or (iii) inner reefs (i.e., nearshore reefs) adjacent to islands. Although some locations had extensive lagoons (i.e., Palau and Pohnpei), other locations did not (i.e., Yap and Kosrae). Majuro and Kiritimati did not have inner reefs (i.e., nearshore reefs), and only had patch reefs (i.e., lagoonal reefs) and outer reefs. Therefore, the number of sites sampled per habitat varied according to the area of available habitat at each location.

Benthic surveys were conducted at each of the six island locations. At each site, six 10-m long fiberglass transect tapes were laid to follow the contour of the reef substrate at a depth between 2–5 m low-water-spring tide. The end of one tape was spaced at least 2 m from the start of the next tape. The line-intercept method was applied along each transect to quantify the planar-chord length of each benthic component to the nearest centimeter. Corals were identified to species level, except encrusting *Montipora* and massive *Porites*, which were identified to life forms. Crustose coralline algae, macroalgae, sponges, ascidians, tunicates, and other benthic components were identified to the highest taxonomic resolution that was possible in the field. Along the same transects all echinoids that were located within 30 cm either side of the tape were counted and identified as either *Diadema*, *Echinometra*, or ‘Other’ urchins. The diameter of each echinoid test was measured to the nearest millimeter. Also, at each site, and directly over each of the six 10-m transect tapes that followed the reef contour, a second 10-m fiberglass transect tape was spanned tightly (from the same starting point as the contour tape). The position of the 10-m mark (i.e., the end) of the first tape along the second tape was recorded to the nearest cm. This difference in length between the two tapes (i.e., between the tightly-spanned-horizontal tape and the contour-following tape) was used to estimate reef rugosity.

In addition to the benthic surveys, each site was surveyed for herbivorous fishes (starting prior to the benthic surveys to ensure that the fishes were not scared away) using a high-definition video camera Canon HFM500 housed in a Canon WP-V4 waterproof housing. Six, 30-m long by 4-m wide belt transects were used to record the fishes at a depth between 2–5 m low-water-spring tide. The length of each transect was determined by one diver of the buddy pair attaching a spool of twine to the reef substrate with a rubber band, and then swimming forward and unravelling the twine to the 30 m mark while their dive buddy was responsible for videoing. The twine was then tugged, to break the rubber band, and then reeled back ready to start the next transect. The width of the video field of view was initially calculated by laying a 4-m tape perpendicular to the transect line. The end of one transect was spaced at least 2 m from the start of the next transect. From the videos, herbivorous parrotfishes were identified to species level [38] and their estimated size was recorded to the nearest centimeter. The calculations to estimate carbonate production and erosion at each site are provided in S1 File.

### Environmental variables

Predictor variables were obtained from satellite observations. El Niño Southern Oscillation (ENSO), Pacific Decadal Oscillation (PDO), Degree Heating Weeks (DHW), and sea-surface temperature signals were obtained from the Coral Reef Temperature Anomaly Database (CoRTAD) (https://data.nodc.noaa.gov/cortad/Version6/), Version 6 with 4-km grid cells [39]. ENSO and PDO signals, including phase combinations, were derived by regressing the sea surface temperature (SST) time series data from “Sea Surface Temperature and Related Thermal Stress Metrics from 2005–2017” from the National Oceanic and Atmospheric Administration (NOAA) climate indices on monthly atmospheric and ocean time series (https://psl.noaa.gov/data/climateindices/). DHW was considered under multiple metrics, as the maximum over the minimum for each cell in the timeframe and as the cumulative and average DHW for the 12-year timeframe. The rate of increase in thermal anomalies was derived from Extended Reconstructed Sea-Surface Temperature (ERSST) v5 (https://www.ncdc.noaa.gov/data-access/marineocean-data/extended-reconstructed-sea-surface-temperature-ersst-v5) as a 2x2 degree grid using the monthly time-series data from 2010–2018 compared with a baseline from 1971–2000. To calculate the frequency of thermal-stress events the weekly data, for a given year, was summed using the number of times over the previous 52 weeks that the thermal-stress anomaly was ≥ 1°C. These values were then averaged over the years 2000–2020. The baseline climatology was generated using a harmonic analysis procedure that fit annual and semi-annual signals to the time series of weekly SSTs at each grid cell [40, 41]. A raster of the frequency of cyclones was obtained from International Best Track Archive for Climate Stewardship (IBTrACS) (https://www.ncdc.noaa.gov/ibtracs/index.php?name=ibtracsdata), using 50 years of consistent sampling effort between 1964 and 2014. The storms were collated based on wind speed following the Saffir-Simpson Scale (SSS) and a heat map was made in QGIS (http://qgis.org) matching the radius of damaging winds (> 26 ms^−1^) to the speed of each cyclone [42]. The summed 50 years of cyclones were then converted to an average number of cyclones per year, per 9.2 km cell yr^-1^ [15].

### Data analysis

Variables were checked for multicollinearity using a Pearson’s correlation, and any variables with a correlation coefficient of more than positive or negative 0.7 were removed (ENSO, cyclone frequency, and PDO were removed from the analysis because ENSO was strongly positively correlated with anomalous SST, cyclone frequency was strongly negatively correlated with anomalous SST, and PDO was strongly negatively correlated with the frequency of thermal stress). We examined the relationship between the percentage live coral cover and net accretion rate for each habitat within each island, across all study sites, using a generalized additive model [43]. Integrated Nested Laplace Approximation (INLA) [44] within a Bayesian framework was used to examine spatial differences in carbonate production, defined as:

$$y_{i}∼N\left(\mu_{i},\sigma \right)$$ (1)

where *y*_i_ represents the vector of net carbonate production at site *i*, *μ* is a vector of expected values, and *σ* is the residual standard deviation, and *N* is a Gaussian distribution. The expected values are represented as:

$$\mu_{i}=\alpha +\sum \beta .Z\left(s_{i}\right)+\xi \left(s_{i}\right)+\varepsilon \left(s_{i}\right)$$ (2)

where α is an intercept coefficient, *β* is the fixed-effect coefficient vector, Z is a matrix of covariates at the location of the data points *s*_i_, ξ(*s*_*i*_) is the spatial random effect in a spatial Gaussian Markov Random Field (GMRF), and ε(*s*_*i*_) is the measurement error defined by a Gaussian white-noise process *~ N* (*0*, *σ*^*2*^_*ε*_). The GMRF combines the Gaussian field with Matérn covariance functions using stochastic partial differential equations, which in turn use a finite element representation to define the Matérn field by triangulation of the spatial domain [45]. This study analyses the coefficients of the covariates *β*, to determine how much of the variance in the carbonate-production data is explained by the covariates. The variation in the spatial effect is explained by the variance-covariance matrix calculated using the Matérn correlation function. This analytical approach is appropriate for our data collected at spatially irregular intervals. Model selection was based on the lowest Deviance Information Criterion (DIC) and the lowest Watanabe-Akaike Information Criterion (WAIC). To validate the model we used spatial leave-one-out cross validation, to assess the root mean squared error [46]. All the R scripts [47] for the calculations and the field data are available at https://github.com/rvanwoesik/Pacific and at: https://www.bco-dmo.org/dataset/736016.

The Palau data were collected under a blanket permit of Yimyang Golbuu the Director of the Palau International Coral Reef Center. The Yap data were collected under the auspices of our collaboration with YapCAP. In Kosrae the data were collected in collaboration with Andy George the Director of the Kosrae Conservation Society (KCS), and the Pohnpei data were collected in collaboration with Eugene Joseph, Director of the Conservation Society of Pohnpei. The Majuro data were collected in collaboration with Glen Joseph, Director of the Majuro Marine Resources Authority (MIMRA) and Emma Kabua-Tibon, Coastal Division Chief of the MIMRA.

## Results

The massive coral *Porites lobata* was a dominant coral at all six locations, and *Porites cylindrica* and *Porites rus* colonies were dominant reef-building corals study wide, except in Kiritimati, which supported mainly *Dipsastraea stelligera* and *Pavona* species (see S1 File). In addition to the three *Porites* species mentioned above, *Acropora formosa* (*muricata*) was a dominant reef-builder in Palau and Yap, and *Acropora palifera* was a dominant reef-builder in Yap, particularly in the shallow lagoon habitat. In Pohnpei and Kosrae, in addition to the afore-mentioned *Porites* species, encrusting *Montipora*, *Acropora hyacinthus*, and *Goniastrea retiformis* were dominant reef-building corals. *Acropora* species dominated the outer reef-building coral assemblages of Majuro, and the afore-mentioned *Porites* species dominated the lagoon (see S1 File).

**Fig 2:**
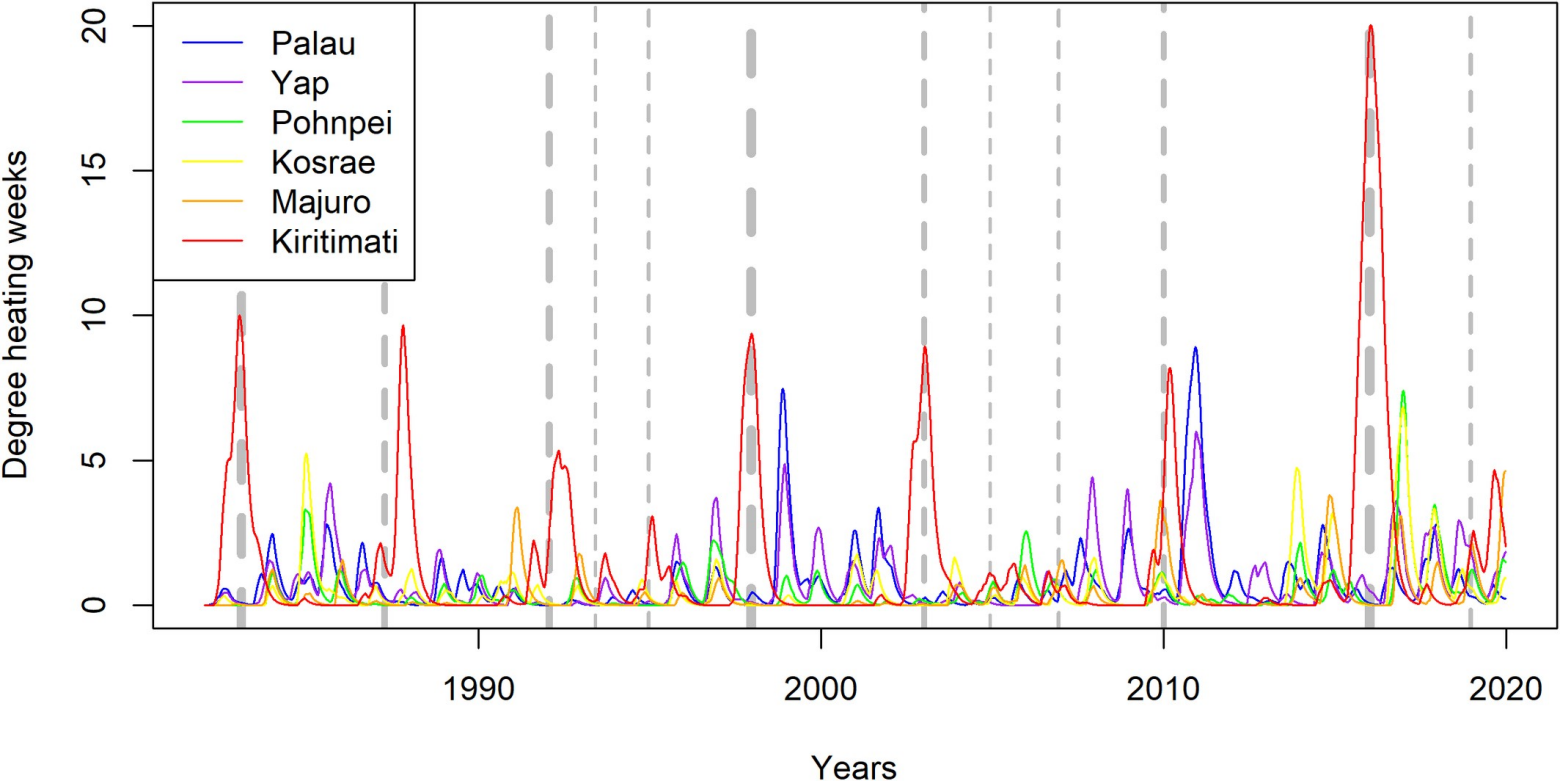
A time series of thermal stress in the Pacific Ocean from 1982 to 2020 at Palau, Yap, Pohnpei, Kosrae, Majuro, and Kiritimati, expressed as degree heating weeks (DHWs), which is the cumulative measure of the intensity and duration of heat stress that exceeds the average summertime maximum temperature (i.e., local climatology) by 1°C, culminated over 12 weeks and expressed in the unit °C-weeks. The timing of El Niño events are indicated with vertical dashed lines and the thickness of the lines are in proportion to the intensity of the event.

In the last four decades, the reefs of Palau and Yap in the western Pacific Ocean have experienced two major thermal-stress events, one during the 1997–98 El Niño and another during the 2010 El Niño (Fig 2). Farther east, the reefs of Pohnpei, Kosrae, and Majuro experienced periodic low-level thermal anomalies during the same period, which were the most intensive during the recent 2015–2017 El Niño (Fig 2). By contrast, the reefs of Kiritimati in the central Pacific Ocean have experienced seven thermal stress events since 1982 (with degree heating weeks above 5) the most extreme of which was from June to November in 2015 when the degree heating weeks were consistently above 15, and sea surface temperatures (SSTs) were up to 5°C above average (Fig 2).

**Fig 3:**
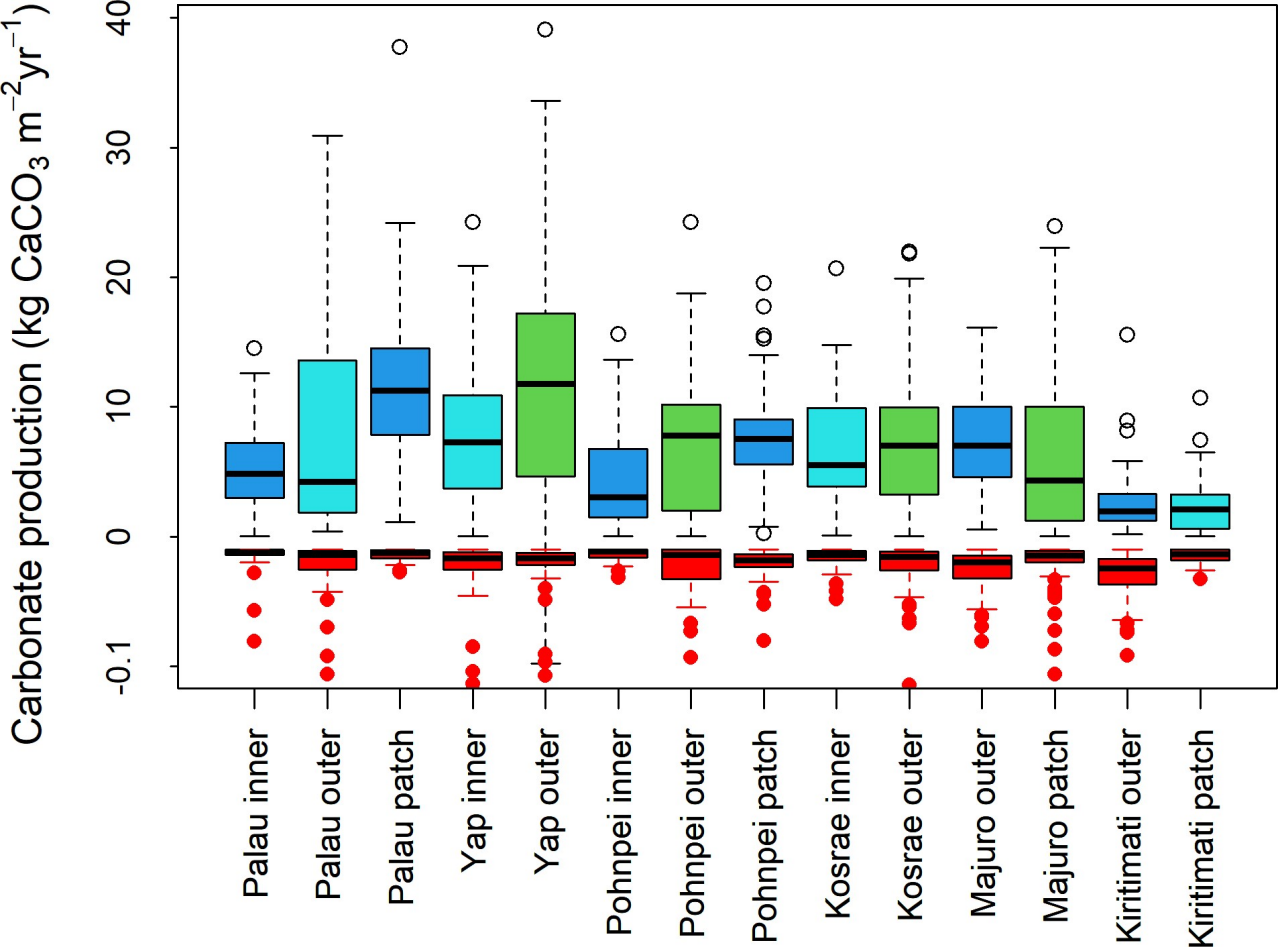
A summary of carbonate production rates (kg CaCO_3_ m^-2^ yr^-1^) in the tropical Pacific Ocean for Palau and Yap (2017), Pohnpei and Kosrae (2018), and Majuro, and Kiritimati (2019), where green represents nearshore reefs, light blue represents patch reefs in lagoons, and dark blue represents outer reefs. The thick horizontal lines are the medians, the boxes surrounding the medians are the first and third quartiles, the whiskers identify the range of the data, and the circles identify the outliers. The red boxes, lines, and circles display the erosion rates.

**Fig 4:**
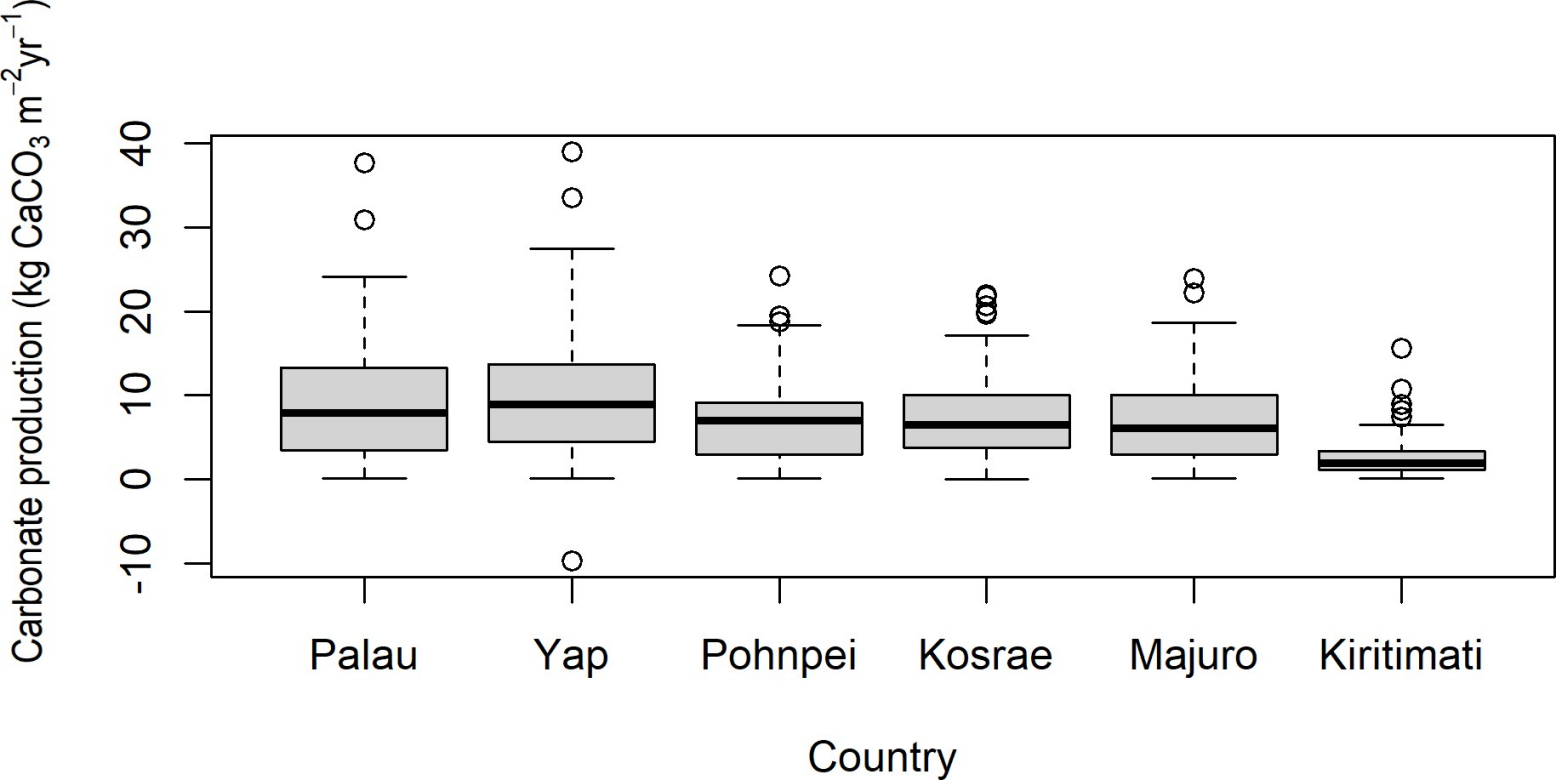
Carbonate production rates (kg CaCO_3_ m^-2^ yr^-1^) of shallow coral reefs in the tropical Pacific Ocean, stratified by island for Palau and Yap (2017), Pohnpei and Kosrae (2018), and Majuro and Kiritimati (2019). The thick horizontal lines are the medians, the boxes surrounding the medians are the first and third quartiles, the whiskers identify the range of the data, and the circles identify the outliers.

**Fig 5:**
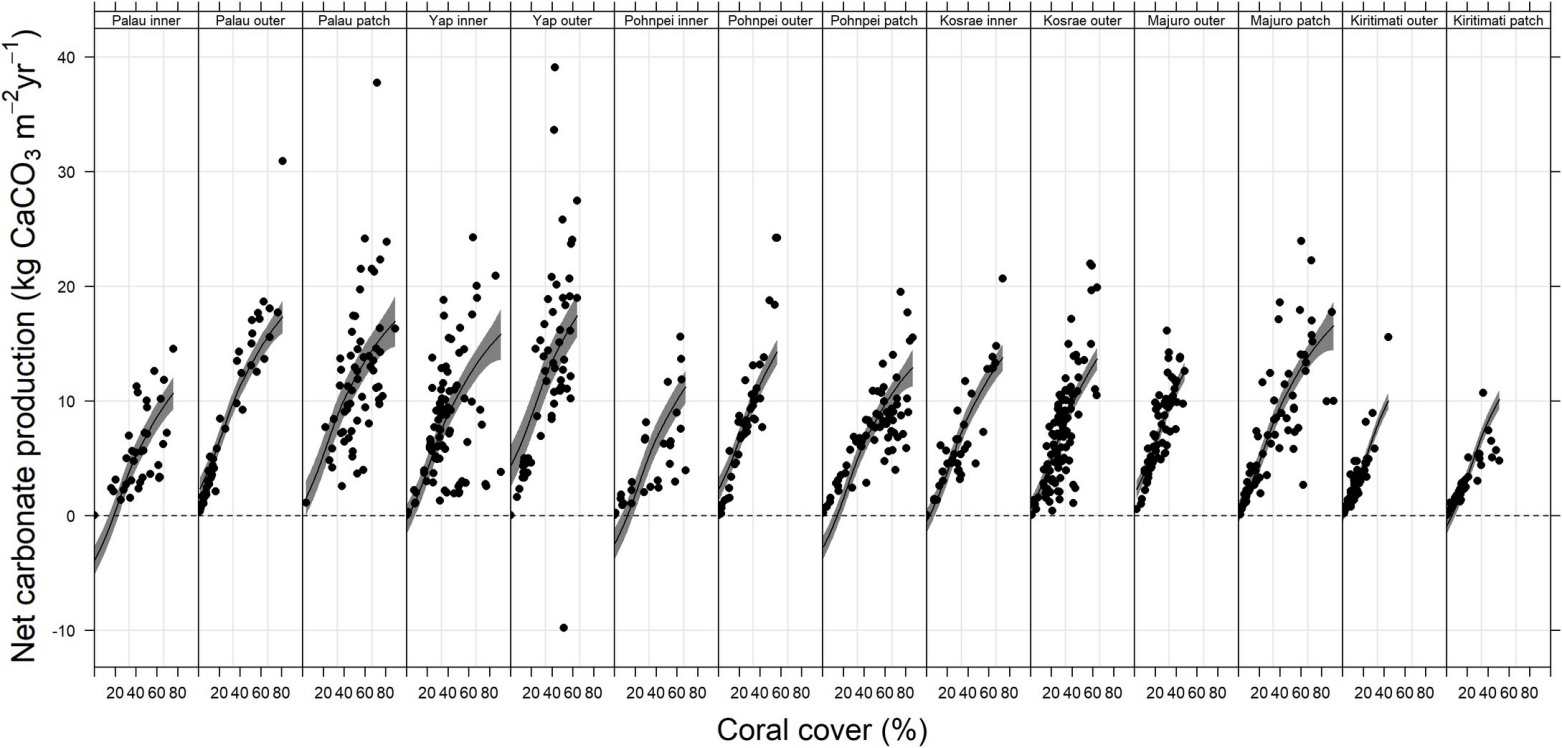
The relationship between the percentage (%) live coral cover and the net carbonate production rates (kg CaCO_3_ m^-2^ yr^-1^) for each habitat (i.e., inner, patch, and outer reefs) at each of the six Pacific Ocean study locations [i.e., at Palau and Yap (2017), Pohnpei and Kosrae (2018), and Majuro and Kiritimati (2019)]. The lines are best-fit generalized additive models, and the polygons display the standard errors.

The history of heat-stress disturbances were reflected in the field estimates of net carbonate production rates. The study’s western-most reefs (around Yap and Palau) had considerably higher than average net carbonate production (~9 kg CaCO_3_ m^-2^ y^-1^) than the reefs located further east (especially those of Kiritimati in the central Pacific Ocean) which averaged (~2.5 kg CaCO_3_ m^-2^ y^-1^) (Fig 3). Several outer reefs of Yap and Palau, particularly the western outer reefs, had particularly high net carbonate production rates, > 15 kg CaCO_3_ yr^-1^ (S1 File). The net carbonate production rates of Pohnpei, Kosrae, and Majuro were moderate (~ 7 kg CaCO_3_ yr^-1^) (Fig 3). In general, the inner reefs (i.e., nearshore reefs) of all four continental islands of Palau, Yap, Pohnpei, and Kosrae recorded the lowest rates of net carbonate production (~6 kg CaCO_3_ m^-2^ y^-1^) in comparison with the other reef habitats [i.e., lagoonal patch reefs and outer reefs] at the same islands (Fig 4 and Table 1). The outer reefs of this study, in general, had higher erosion rates than the patch reefs and inner reefs (Fig 4), primarily driven by the abundance of large parrotfishes (S1 File). Importantly, the value of live coral cover at which net carbonate production became negative was highest on the inner reefs of this study (Fig 5), suggesting that inner reefs require more coral cover to produce the same carbonate as outer reefs.

**Table 1:** Posterior marginal distributions of fixed effects of the best-fit model for carbonate production rates and environmental covariates across the tropical Pacific Ocean at Palau and Yap (2017), Pohnpei and Kosrae (2018), Majuro and Kiritimati (2019) using integrated nested laplace approximation.

	Mean	Standard deviation	2.5% CI	97.5% CI
**Intercept**	2.562	0.704	1.070	3.976
**SST anomaly**	-1.837	0.981	-3.942	0.019
**SST Frequency**	-0.097	0.647	-1.424	1.146
**Outer Reefs**	2.437	0.992	0.488	4.384
**Patch reefs**	2.741	1.152	0.509	5.036
SST Anomaly*SST Frequency	-0.7813	0.644	-1.942	0.610

Where CI indicates credible intervals, SST is the sea-surface temperature, the Deviance Information Criterion (DIC) = 818.53, the Watanabe-Akaike information criterion (WAIC) = 819.31, the marginal log-Likelihood = -439.67, and the * indicates multiplication.

**Fig 6:**
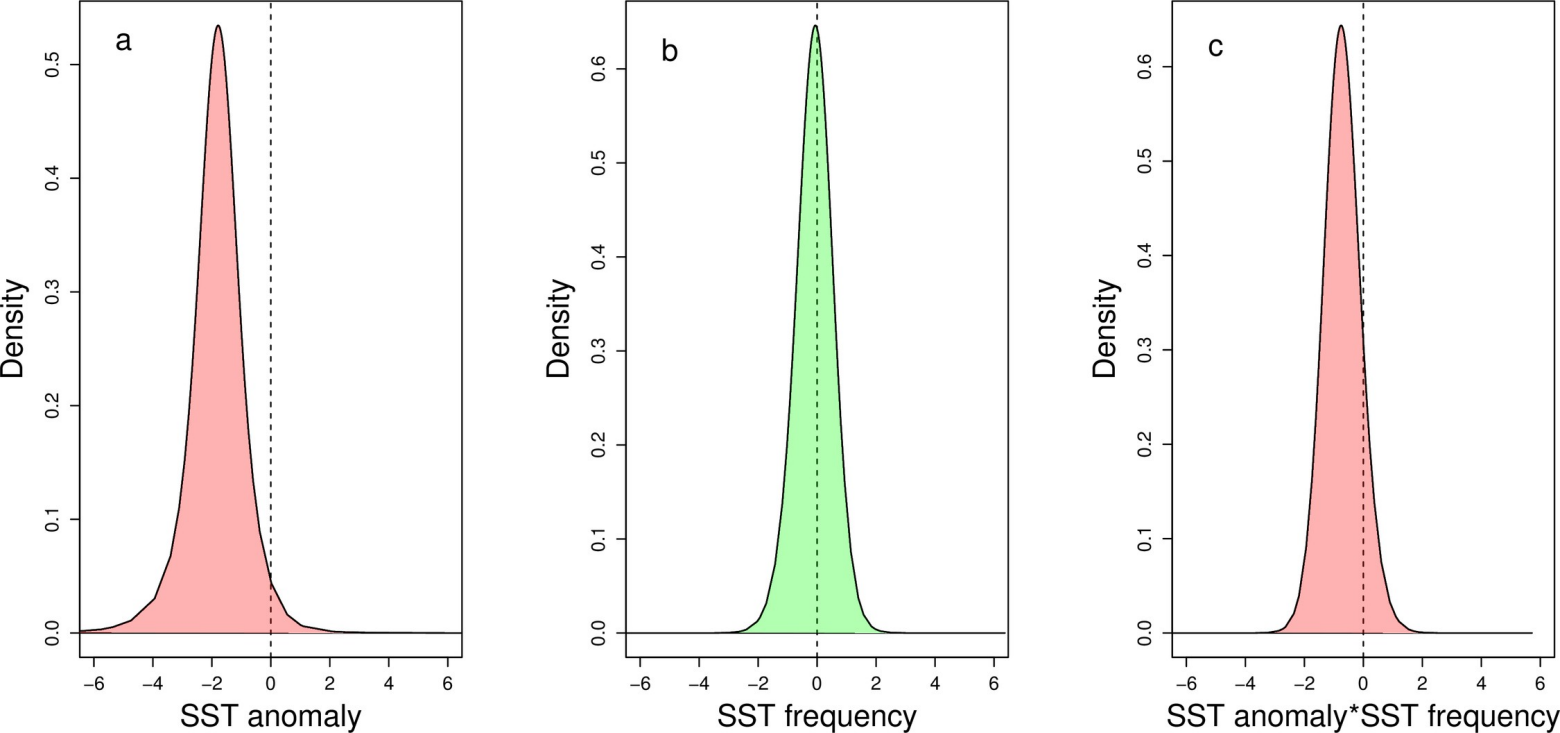
Posterior marginal distributions of fixed effects in the integrated nested laplace approximation model for: (a) sea surface temperature (SST) anomalies, (b) return frequency of SST, and (c) the interaction between SST anomalies and return frequency of SST averaged from 6 January 2000 to 26 December 2019 at each of the six Pacific Ocean study locations [i.e., at Palau and Yap (2017), Pohnpei and Kosrae (2018), and Majuro and Kiritimati (2019)]. Where the dashed lines represent zero, the pink shading represents negative effects, the green shading represents neutral effects, and the * indicates multiplication.

**Fig 7:**
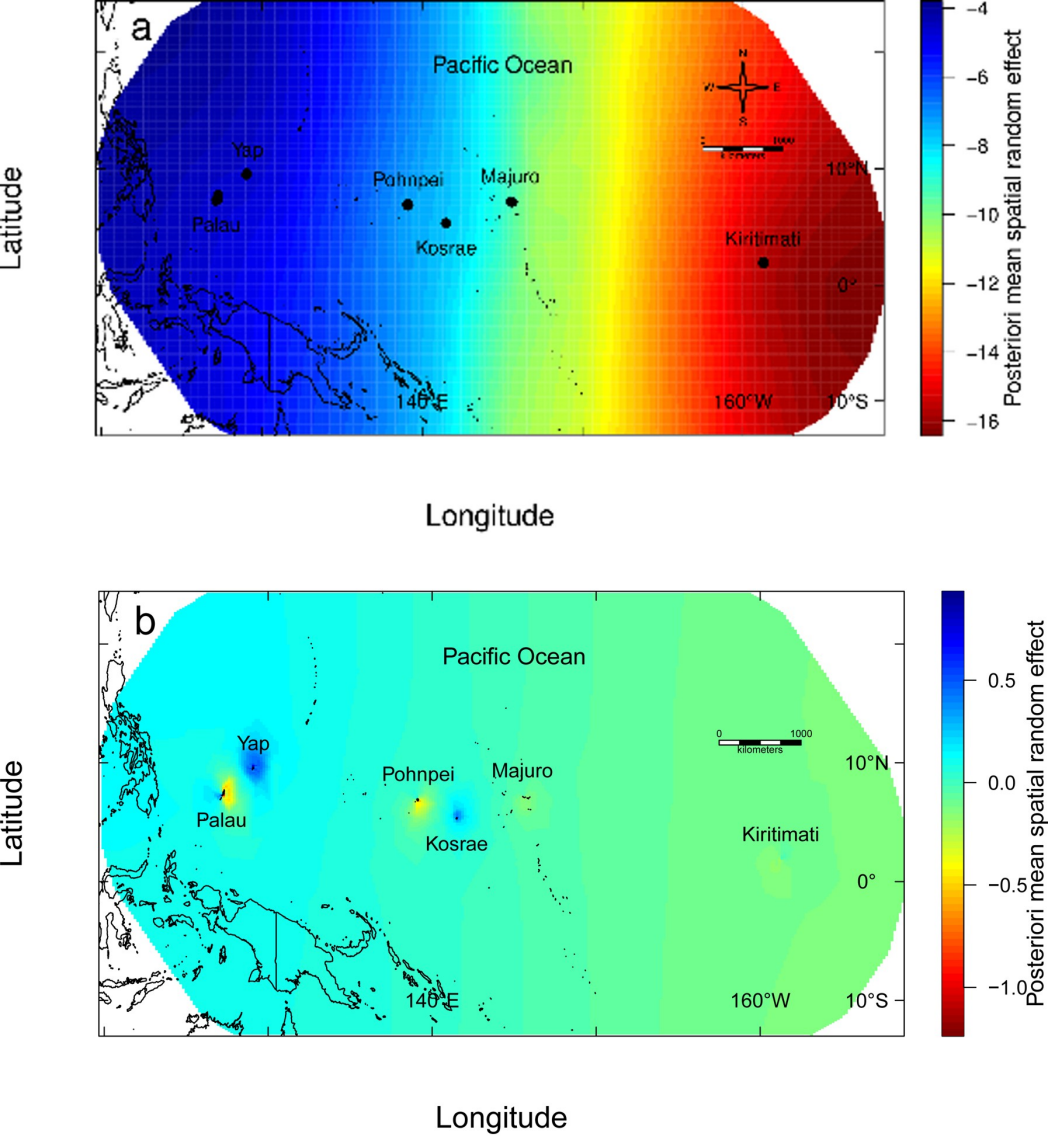
Posterior of mean spatial random effect (a) without and (b) with the frequency of return period thermal-stress covariate incorporated in the Integrated Nested Laplace Approximation model averaged from 6 January 2000 to 26 December 2019 at each of the six Pacific Ocean study locations [i.e., at Palau and Yap (2017), Pohnpei and Kosrae (2018), and Majuro and Kiritimati (2019)]. Outline of land plotted from the R package ‘rworldmap’ [64].

The model with the lowest Deviance Information Criterion (DIC) and the lowest Watanabe-Akaike Information Criterion (WAIC) included the covariates anomalous SST, return frequency of anomalous SST, habitat type, and an interaction between anomalous SST and return frequency of anomalous SST as fixed factors, and included a spatial random field (Table 1). Notably, the intensity of anomalous SST and the interaction between anomalous SST and the return frequency of anomalous SST both had negative effects on net carbonate production (Fig 6), although the return frequency of SST anomalies alone did not have a noticeable effect on net carbonate production. When the return frequency of anomalous SST was excluded from the model, there was a strong latent spatial trend across the Pacific Ocean (Fig 7A), which was less defined when the return frequency of anomalous SST was included in the model (Fig 7B). There was a latent spatial effect, not explained by thermal stress, across the Pacific. There were also localized latent spatial effects of low net carbonate production along the eastern outer reefs of Palau, explained by the overpass of two recent typhoons, and along the southeastern outer reefs of Pohnpei (Fig 7B). Using the spatial leave-one-out cross validation approach, our model had a root mean squared error of 4.9 (confidence intervals, 3.8, 7.2) (Fig T in S1 File).

## Discussion

Over the last four decades, the western reefs in this study region have experienced less thermal stress than the central Pacific reefs. In particular, the reefs of Kiritimati suffered considerable loss in coral cover during the thermal stress associated with the 2014–2017 El Niño when, from June to November 2015, sea surface temperatures (SSTs) were up to 5°C above average [48, 49]. These thermal-stress events are reflected in the rates of net carbonate production estimated on the reefs from 2017 to 2019. Indeed, the past intensity of thermal-stress events was a strong predictor of the rates of net carbonate production across the Pacific, with the reefs of Palau and Yap, in the western Pacific, showing considerably higher average rates of net carbonate production than the reefs of Kiritimati in the central Pacific.

In general, the inner reefs (i.e., the nearshore reefs) closest to islands, tended to produce less carbonate than patch reefs (i.e., lagoonal reefs) and outer reefs. These inner reefs therefore need a higher percentage of coral cover to produce the same amount of carbonate as patch and outer reefs. The lower rates of net carbonate production nearshore were not necessarily a consequence of greater erosion rates nearshore. Our study found that the nearshore reefs in general had lower external erosion rates than outer reefs (Fig 3), primarily a consequence of fewer and smaller parrotfishes nearshore than on outer reefs (S1 File). This study did not measure internal erosion rates, which are known to be relatively higher nearshore than on outer reefs [50]. The differences in the rates of net carbonate production between inner and outer reefs seem to stem primarily from differences in species composition and coral colony morphologies, because we used the same average coral growth rates for each species across habitats, although environmental conditions that differentially influence coral community structure among habitats may contribute to additional variability.

Across the region, there were more acroporids (i.e., corymbose *Acropora* and encrusting *Montipora*) and pocilloporids on outer reefs than in lagoons and on nearshore reefs. There were also more massive, branching, and columnar *Porites* (i.e., *Porites lobata*, *P*. *cylindrica*, and *P*. *rus*) nearshore and on lagoonal reefs than on outer reefs. Although the annual rates of growth between *Porites* and *Acropora* are substantial [51], Roff [52] recently reported no relationship between growth rates of coral species and rates of reef accretion. In other words, reefs that supported massive *Porites* colonies accreted just as quickly as reefs that supported *Acropora* colonies [52]. There are however considerable differences in environmental conditions across nearshore to offshore gradients. For example, the nearshore reefs of Palau have a 1.5-fold greater light extinction coefficient than outer reefs [53]. Nearshore reefs of Palau also experience higher sedimentation rates than outer reefs [54], which can reduce juvenile and adult coral-colony densities and coral cover [10]. Irrespective of the reason why nearshore reefs produce less carbonate than outer reefs, these results suggest considerable vulnerability of nearshore reefs. These results stress the need for effective land-use practices and management at the local level as the climate continues to change.

There were over 100 coral species contributing to carbonate production in the surveyed transects at Palau and Yap (in the western Pacific) and Pohnpei, Kosrae, and Majuro (further to the east), yet less than 10% of the coral species contributed upwards of 75% of the reef’s carbonate production (see S1 File). By contrast, there were fewer than 50 coral species contributing to carbonate production in the surveyed transects at Kiritimati (in the central Pacific), but again 10% of those coral species contributed upwards of 75% of the reef’s carbonate production (see S1 File). Interestingly, while many of the main reef-building corals were common across all islands, such as *Porites lobata*, a few species that contributed to the majority of carbonate production differed for each island. Even islands that were geographically adjacent, had some dominant reef-building corals that differed. For example, *Acropora formosa* (*muricata*) was a major contributor of carbonate production in Palau but not in Yap, whereas *Acropora palifera* was a major contributor of carbonate production in Yap but less so in Palau. Similarly, *Porites cylindrica* was a major contributor of carbonate production in Pohnpei but not Kosrae, whereas *Porites lichen* was a major contributor of carbonate production in Kosrae and not in Pohnpei. Geographic circumstance and chance events may afford an advantage of one dominant coral species over another, although essentially playing a similar role in reef building across similar habitats on different islands.

Such interchangeability and substitutions of coral species in carbonate production suggests some functional redundancy across the western tropical Pacific Ocean, with different dominant coral species capable of producing similar rates of carbonate production, although these redundancies may be only effective over the long-term. In the short-term, after thermal-stress events, such substitutions are less likely. Asymmetrical contributions, with only a few coral species contributing to most of the carbonate production, might be a sign of vulnerability if these dominant reef-building coral species locally decline. Such reductions in the capacity to produce carbonate through loss of coral after thermal stress was most evident in Kiritimati. The dominant coral assemblages of Kiritimati were relatively unusual for Pacific reefs, in part because of their relative isolation [55, 56], but also because they are remnant assemblages from the 2015 thermal-stress event [49]. Increased thermal stress combined with the isolation of central Pacific reefs poses a serious threat to the ability of these reefs to recover rapidly enough from repetitive thermal-stress to keep up with sea-level rise. For example, losing the majority of *Acropora* reef-building colonies in the Caribbean has had similar consequences and has impeded contemporary net carbonate production throughout the Caribbean [11].

Our study also identified a localized latent effect along the eastern offshore reefs of Palau in the best-fit model (Fig 7B), which is related to the destruction caused to the eastern outer reefs by Typhoon Bopha in 2012 and Typhoon Haiyan in 2013 [57]. Low latitude cyclones are unusual, but are becoming more common with climate change [58]. Our analyses identified that the return frequency of SST decreased toward the Coral Triangle (Fig 7), but also identified a latent spatial trend across the Pacific Ocean that was not explained by thermal stress. We hypothesize that this latent trend may be related to the proximity of the Coral Triangle, which may intermittently enhance coral recovery through larval supply [59]. The reefs of the Coral Triangle, which extend from the Philippines in the north through western Indonesia, to Timor in the south and the Solomon Islands in the east, include reefs with relatively high percentages of coral cover [9, 60]. These reefs can supply frequent and massive pulses of larvae to nearby regions [59]. Pacific reefs near the Coral Triangle have also had less coral bleaching than elsewhere in the Pacific over the last two decades [9]. Therefore, the interaction between reduced thermal stress and a high larval supply [59] may have some consequences on reef provisioning on reefs adjacent to the Coral Triangle.

Although the rates of net carbonate production across the western and central study sites (i.e., Palau, Yap, Pohnpei, and Kosrae) were relatively high (i.e., 8.2 kg CaCO_3_ m^-2^ y^-1^) compared with rates in the modern Caribbean (~1.5 kg CaCO_3_ m^-2^ y^-1^, [11]), it is very likely that the rates of net carbonate production will diminish in the future, with increasing frequency and intensity of thermal anomalies under climate change [61]. Averaging across the reef sites and habitats of Palau and Yap (in the western region of this study) in the western Pacific, the rate of net carbonate production equated to approximately 7–8 mm y^-1^ of potential vertical accretion (after [28]). We add that these vertical accretion rates are classified as potential accretion rates, because other processes also play a role in reef accretion, including reef porosity, terrigenous and calcareous sedimentation rates, physical breakage, water-flow rates, and cementation, which we did not measure. Still, such potential rates of vertical accretion that we estimated are higher than projected averages of sea-level rise for the representative concentration pathway (RCP) climate-change scenarios 2.6, 4.5, and 6, but lower than the RCP scenario 8.5 [5]. By contrast, the lower rates of net carbonate production of Kiritimati (in the eastern region of this study) in the central Pacific, were approximately 2 mm y^-1^ of potential vertical accretion. Such rates of vertical accretion are lower than projected average rates of sea-level rise for the representative concentration pathway (RCP) climate-change scenarios 4.5, and 6, and 8.5. Averaging across the reef sites and habitats of Pohnpei, Kosrae, and Majuro (in the mid region of this study), the rate of net carbonate production (~8.2 kg CaCO_3_ m^-2^ y^-1^) equated to approximately 6 mm y^-1^ of potential vertical accretion, which is higher than the RCP scenarios 2.6 and 4.5, but lower than the RCP scenarios 6 and 8.5.

In conclusion, the net rates of carbonate production on shallow reefs in the western tropical Pacific Ocean were considerably higher than the shallow reefs in the central tropical Pacific Ocean, which experienced coral bleaching in 2015 during a major thermal-stress event related to the 2014–2017 El Niño. The low net carbonate production rates of Kiritimati reefs (in the eastern region of this study), in the central Pacific Ocean, equated to approximately ~2 mm y^-1^ of potential vertical accretion. Such rates are too low to keep up with projected sea-level rise for all representative concentration pathways (RCPs), except possibly RCP 2.6.

The results also show that in general inner reefs (i.e., nearshore reefs) do not produce as much carbonate as patch reefs (i.e., lagoonal reefs) and outer reefs. Nearshore reefs are closest to detrimental land-use practices, which may make them more vulnerable to sea-level rise than other habitats. These results stress the need to protect nearshore reefs from local pollutants and the need for effective land-use practices as the oceans continue to warm. Although sea-level rise will provide some accommodation space to shallow coral reefs, with an opportunity for *Porites* microatolls to commence growing vertically [62] and for coral colonization on barren reef flats—the predicted increase in the intensity and frequency of thermal-stress events, however, will seriously jeopardize net carbonate production and impede the capacity of coral reefs to keep up with sea-level rise. If reefs lose the capacity to keep up with sea-level rise, island nations that rely on reefs as habitat for critically important wave barriers are threatened, especially as sea-level rise accelerates and ocean temperatures continue to increase.

## Supporting information

S1 File Twenty supporting figures and one supporting table. **Fig A.** Spatial kriging of the net shallow-water coral-reef carbonate production (kg CaCO_3_ m^-2^ yr^-1^) from 24 sites in Palau, 2017. **Fig B.** Contribution of carbonate production (kg CaCO_3_ m^-2^ y^-1^) by coral species, from 24 sites in Palau, 2017. Panel **a)** shows the contribution of species across all 24 sites, panel **b)** shows the contribution of species across the 10 patch reef sites, panel **c)** shows the contribution of species across the 8 outer reef sites and panel **d)** shows the contribution of species across the 6 inner reef sites. **Fig C.** Erosional estimates for parrotfishes (kg CaCO_3_ m^-2^ yr^-1^) using spatial kriging for shallow-water coral-reef habitats (including inner, outer, and patch reefs), from 24 sites in Palau, 2017. **Fig D.** Spatial kriging of the net shallow-water coral-reef carbonate production (kg CaCO_3_ m^-2^ yr^-1^) from 24 sites in Yap, 2017. **Fig E.** Contribution of carbonate production (kg CaCO_3_ m^-2^ y^-1^) by coral species from 24 sites in Yap, 2017. Panel **a)** shows the contribution of species across all 24 sites, panel **b)** shows the contribution of species across the 14 inner reef sites, and panel **c)** shows the contribution of species across the 10 outer reef sites. **Fig F.** Erosional estimates for parrotfishes (kg CaCO_3_ m^-2^ yr^-1^) using spatial kriging for shallow-water coral-reef habitats (including inner, outer, and patch reefs) from 24 sites in Yap, 2017. **Fig G.** Spatial kriging of the net shallow-water coral-reef carbonate production (kg CaCO_3_ m^-2^ yr^-1^) from 24 sites in Pohnpei, 2018. **Fig H.** Contribution of carbonate production (kg CaCO_3_ m^-2^ y^-1^) by coral species from 24 sites in Pohnpei, 2018. Panel **a)** shows the contribution of species across all 24 sites, panel **b)** shows the contribution of species across the 11 patch reef sites, panel **c)** shows the contribution of species across the 8 outer reef sites and panel **d)** shows the contribution of species across the 5 inner reef sites. **Fig I.** Erosional estimates for parrotfishes (kg CaCO_3_ m^-2^ yr^-1^) using spatial kriging for shallow-water coral-reef habitats (including inner, outer, and patch reefs) from 24 sites in Pohnpei, 2018. **Fig J.** Spatial kriging of the net shallow-water coral-reef carbonate production (kg CaCO_3_ m^-2^ yr^-1^) from 24 sites in Kosrae, 2018. **Fig K.** Contribution of carbonate production (kg CaCO_3_ m^-2^ y^-1^) by coral species from 24 sites in Kosrae, 2018. Panel **a)** shows the contribution of species across all 24 sites, panel **b)** shows the contribution of species across the 6 inner reef sites, and panel **c)** shows the contribution of species across the 18 outer reef sites. **Fig L.** Erosional estimates for parrotfishes (kg CaCO_3_ m^-2^ yr^-1^) using spatial kriging for shallow-water coral-reef habitats (including inner, outer, and patch reefs) from 24 sites in Kosrae, 2018. **Fig M.** Spatial kriging of the net shallow-water coral-reef carbonate production (kg CaCO_3_ m^-2^ yr^-1^) from 24 sites in Majuro, 2019. **Fig N.** Contribution of carbonate production (kg CaCO_3_ m^-2^ y^-1^) by coral species from 24 sites in Majuro, 2019. Panel **a)** shows the contribution of species across all 24 sites, panel **b)** shows the contribution of species across the 13 patch reef sites, and panel **c)** shows the contribution of species across the 11 outer reef sites. **Fig O.** Erosional estimates for parrotfishes (kg CaCO_3_ m^-2^ yr^-1^) using spatial kriging for shallow-water coral-reef habitats (including outer, and patch reefs) from 24 sites in Majuro, 2019. **Fig P.** Spatial kriging of the net shallow-water coral-reef carbonate production (kg CaCO_3_ m^-2^ yr^-1^) from 22 sites in Kiritimati, 2019. Note that the eastern reefs of Kiritimati were only lightly surveyed because of inclement ocean conditions. **Fig Q.** Contribution of carbonate production (kg CaCO_3_ m^-2^ y^-1^) by coral species from 22 sites in Kiritimati, 2019. Panel **a)** shows the contribution of species across all 22 sites, panel **b)** shows the contribution of species across the 8 patch reef sites, and panel **c)** shows the contribution of species across the 14 outer reef sites. **Fig R.** Erosional estimates for parrotfishes (kg CaCO_3_ m^-2^ yr^-1^) using spatial kriging for shallow-water coral-reef habitats (including inner, outer, and patch reefs) from 22 sites in Kiritimati, 2019. Note that the eastern reefs of Kiritimati were only lightly surveyed because of inclement ocean conditions. **Fig S.** Generalized additive model examining the relationship between live coral cover and net carbonate production at all 142 sites across the western and central tropical Pacific Ocean. Where the shading represents the 95% confidence intervals, s represents the smoothing function, and the rug plots represent the raw data. Twenty-four of the 142 sites were located in the western Pacific Ocean at each of Palau and Yap in 2017, 24 sites were located progressively further east at each of Pohnpei and Kosrae in 2018, and at Majuro in 2019, and 22 sites were located in the central Pacific Ocean at Kiritimati in 2019. **Fig T.** Spatial cross-validation using spatial leave-one-out cross validation, comparing the observed response of carbonate production against the predicted response of carbonate production using INLAutils (Lucas et al. 2020) for all 142 sites across the western and central tropical Pacific Ocean, and where the identity function (i.e., y = x) is depicted by the line. **Table 1**: Site locations and corresponding model output mean and standard deviation (SD) including Live Coral Cover (LCC), gross production, net production, bioerosion and rugosity.
(PDF)

S1 Data Data and R code. Spreadsheet data for each site of the 142 sites across the Pacific Ocean, and all the R scripts that produced the manuscript figures.
(ZIP)
