# Supplementary material for: Thermal stress jeopardizes carbonate production of coral reefs across the western and central Pacific Ocean
Source: PLoS One. 2021 Apr 26;16(4):e0249008. doi: 10.1371/journal.pone.0249008 (PMC8075252; doi:10.1371/journal.pone.0249008)
Supplement: S1 File — Fig A. Spatial kriging of the net shallow-water coral-reef carbonate production (kg CaCO3 m-2 yr-1) from 24 sites in Palau, 2017. Fig B. Contribution of carbonate production (kg CaCO3 m-2 y-1) by coral species, from 24 sites in Palau, 2017. Panel a) shows the contribution of species across all 24 sites, panel b) shows the contribution of species across the 10 patch reef sites, panel c) shows the contribution of species across the 8 outer reef sites and panel d) shows the contribution of species across the 6 inner reef sites. Fig C. Erosional estimates for parrotfishes (kg CaCO3 m-2 yr-1) using spatial kriging for shallow-water coral-reef habitats (including inner, outer, and patch reefs), from 24 sites in Palau, 2017. Fig D. Spatial kriging of the net shallow-water coral-reef carbonate production (kg CaCO3 m-2 yr-1) from 24 sites in Yap, 2017. Fig E. Contribution of carbonate production (kg CaCO3 m-2 y-1) by coral species from 24 sites in Yap, 2017. Panel a) shows the contribution of species across all 24 sites, panel b) shows the contribution of species across the 14 inner reef sites, and panel c) shows the contribution of species across the 10 outer reef sites. Fig F. Erosional estimates for parrotfishes (kg CaCO3 m-2 yr-1) using spatial kriging for shallow-water coral-reef habitats (including inner, outer, and patch reefs) from 24 sites in Yap, 2017. Fig G. Spatial kriging of the net shallow-water coral-reef carbonate production (kg CaCO3 m-2 yr-1) from 24 sites in Pohnpei, 2018. Fig H. Contribution of carbonate production (kg CaCO3 m-2 y-1) by coral species from 24 sites in Pohnpei, 2018. Panel a) shows the contribution of species across all 24 sites, panel b) shows the contribution of species across the 11 patch reef sites, panel c) shows the contribution of species across the 8 outer reef sites and panel d) shows the contribution of species across the 5 inner reef sites. Fig I. Erosional estimates for parrotfishes (kg CaCO3 m-2 yr-1) using spatial kriging for [file pone.0249008.s001.pdf]

Supplementary document for:

Carbonate production of coral reefs across the western and central Pacific Ocean

R. van Woesik and C. Cacciapaglia

Extended methods

The primary task of this work was to determine the contribution of the various components on each reef to potential carbonate production. Coral cover was calculated as the sum of live coral cover for each transect. Net carbonate production (kg $CaCO_3$ m^{-2} yr^{-1}) was considered as:

$$Carbonate\ production_i = Cal_i + sgn(x)Sed_i - Eros_i \qquad (1),$$

where *Cal* is the rate of calcification by reef-building corals and coralline algae, at a site *i*, sgn is positive when local sedimentation (*Sed*) is low, and negative when local sedimentation is high, and *Eros* is the rate of erosion (van Woesik and Cacciapaglia 2018, 2019). Gross carbonate production was estimated in units of kg $CaCO_3$ m^{-2} yr^{-1}, and was summed across all calcifying species of reef accretors, where *Cal* was estimated as:

$$Cal_i = r_i * \{\Sigma[(m_{i,j} * x_{i,j} / 100) * d_{i,j} * g_{i,j} * 10] + ca_i\} \qquad (2),$$

where *r* is the averaged rugosity of site *i*, *m* is the morphological adjustment coefficient for coral morphologies at site *i* for species *j*, *x* is the mean percent planar cover of carbonate-accreting species *j* at site *i*, *d* is the density (g cm^{-3}) of species *j* at site *i*, and *g* is the vertical growth rate (cm $year^{-1}$) of species *j* at site *i*. Ten was inserted in the model as an adjustment coefficient to set the units at kg $CaCO_3$ m^2 yr^{-1}, and *ca* is the contribution of coralline algae at site *i* to carbonate production, which was defined as:

$$ca_i = 0.018 * (pca_i) * 10 \qquad (3),$$

where *pca* is the planar cover of coralline algae at site *i*, 0.018 is the average gross carbonate production of coralline algae (g cm^{-2}) (Perry et al. 2012), and 10 is the conversion between g cm^{-2} and kg m^{-2}.

Reef erosion was broken down into three major components, defined as:

$$Eros_i = \Sigma(parrotfish_{i,j} + urchin_{i,j}) + macroboring \qquad (4),$$

where *parrotfish* is the biological erosion caused by parrotfish at site *i* by species *j*, *urchin* is erosion caused by sea urchins at site *i* by species *j*, and *macroboring* is the erosion caused by macroboring organisms. The erosion caused by parrotfish was defined as:

$$parrotfish_i = \Sigma\ (vol_{j,i,n} * sp_{i,j,n} * br_{i,j,n}) * D_i * 365 * 0.001 \qquad (5),$$

where *vol* is the bite volume (cm^3) for individual *n* of species *j* at site *i*, *sp* is the proportion of bites that leave a scar at site *i* for individual *n* of species *j*, *br* is the bite rate (bites day^{-1}) at site *i* of species *j* for individual *n*, *D* is the average density of corals at site *i*, 365 is used to convert erosion rate to years, and 0.001 is to convert g to kg. In equation 5, *vol* was defined as:

$$vol_{i,j,n} = \frac{e^{1.32+0.06 * \text{length}_{i,j,n}}}{1000} \qquad (6),$$

where, *length* is the length (cm) of parrotfish *n* of species *j* in site *i*, the constants 1.32 and 0.06 were generated from a regression of data from Ong & Holland (2010), and 1000 was used to

convert from mm^3 to cm^3. In equation 5, *sp* is the scar proportion of fish *n* of species *j* at site *i*, defined as:

$$sp_{i,j,n} = 1/[1 + e^{-(-2.46 + 0.089 * length_{i,j,n})}] \quad (7),$$

following a regression from data gathered from Bonaldo & Bellwood (2008), where *length* is the length (cm) of fish *n* of species *j* at site *i*. In equation 5, *br* is the bite rate (bites day^{-1}) at site *i* of species *j* for individual *n*, defined as:

$$br_{i,j,n} = 60\left\{\left[\left(4.31 + brc_{i,j} - 0.36\right) - \left(0.045 * reeftime * length_{i,j,n}\right)\right]\right\} \quad (8),$$

where *brc* is the bite rate constant derived from data provided by Peter Mumby (pers. comm.) for species *j* at site *i*, *reeftime* is the length of time fishes spend grazing on the reef estimated at 9 hours a day, *length* is the length (cm) of fish *n* of species *j* at site *i*, 60 is to convert the units from minutes to hours, and all other constants were derived from bite rate data. The bioerosion (kg $CaCO_3$ m^{-2}) caused by echinoids was defined as:

$$urchin_i = \Sigma(Diadema_{i,n} + Echinometra_{i,n} + Other\ urchins_{i,n}) \quad (9),$$

where *Diadema* is the erosion caused by species in the genus *Diadema* at site *i* for individual *n*, *Echinometra* is the erosion caused by species within the genus *Echinometra* at site *i* for individual *n*, *Other urchins* is the erosion caused by echinoid species not in *Echinometra* or *Diadema*. *Diadema* was defined by a function from Januchowski-Hartley et al. (2017) as:

$$Diadema_{i,n} = \left(0.000001 * diameter_{i,n}{}^{3.42}\right) * 0.365 * 0.57 \quad (10),$$

where *diameter* is the diameter (cm) of the *Diadema* test. The function for *Echinometra* follows an equation from Januchowski-Hartley et al. 2017 and was defined as:

$$Echinometra_{i,n} = \left(0.0004 * diameter_{i,n}{}^{1.98}\right) * 0.365 * 0.57 \quad (11),$$

where *diameter* is the diameter (cm) of the *Echinometra* test. *Other urchins* also follows an equation from Januchowski-Hartley et al. (2017) and was defined as:

$$Other\ urchins_{i,n} = \left(0.0001 * diameter_{i,n}{}^{2.32}\right) * 0.365 * 0.57 \quad (12),$$

where *diameter* is the diameter (cm) of the echinoid test. We were particularly interested in the capacity of clinoid sponges to bioerode carbonate substrate, whereas other macroborers such as polychaetes, crustaceans, sipunculids, and molluscs (Glynn 1997) were more inconspicuous during our surveys. Therefore, *macroboring* was defined as:

$$macroboring_i = plamc_i * mec \quad (13),$$

where *plamc* is the mean planar cover of macroboring organisms for site *i*, and *mec* is a macroboring erosion constant, for which we use a conservative estimate of 10 kg $CaCO_3$ m^{-2} y^{-1} for clinoid sponges. We estimated that the positive contribution of sediment to carbonate production was no more than 0.4 kg $CaCO_3$ m^{-2} y^{-1} (Hubbard 1997).

We estimated carbonate production by solving equations (1) to (13) for each transect, and plotted the estimated rates of carbonate production across the spatial fields. We firstly used semivariograms to estimate the extent of spatial autocorrelation, and examined the spatial data for isotrophy (i.e., directionality). We then used the information from the semivariograms, and the isotrophy, to run a series of ordinary kriging analyses to interpolate the data across the spatial fields of both islands. To convert rates of carbonate production to vertical reef growth we used:

$$Vertical\ reef\ growth = Cp + Cp(Cp * alpha) \quad (14),$$

where *Cp* is carbonate production and *alpha* is an estimated coefficient (van Woesik and Cacciapaglia 2018).

Palau

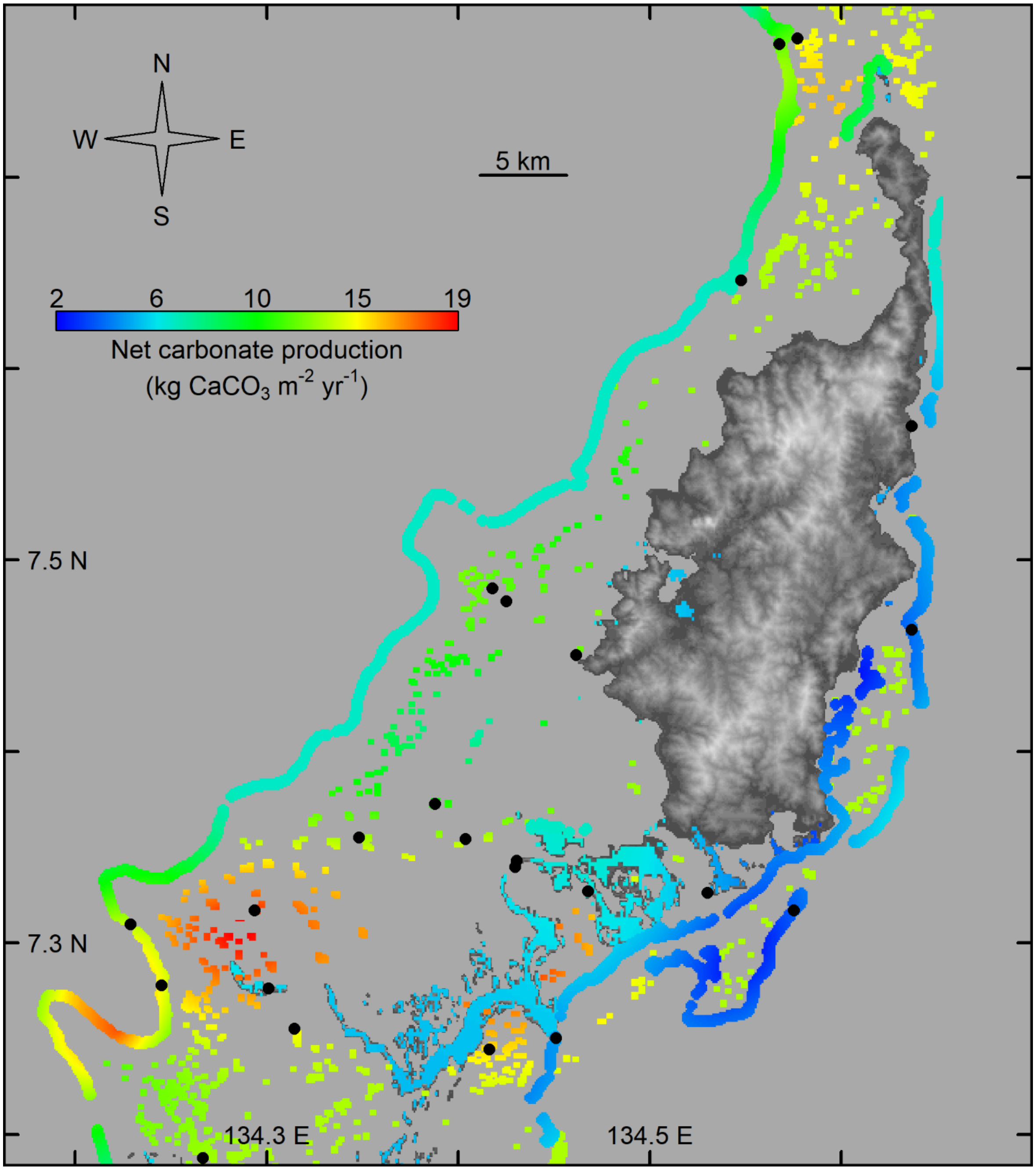

Figure A. Spatial kriging of the net shallow-water coral-reef carbonate production (kg $CaCO_3$ m^{-2} yr^{-1}) from 24 sites in Palau, 2017. The kriged maps were generated in R (R Core Team, 2020) and the topography was generated using 'elevatr' in R (Hollister et al. 2020) using the source GL3 for OpenTopography's data from the Shuttle Radar Topography Mission GL3, https://portal.opentopography.org/raster?opentopoID=OTSRTM.042013.4326.1

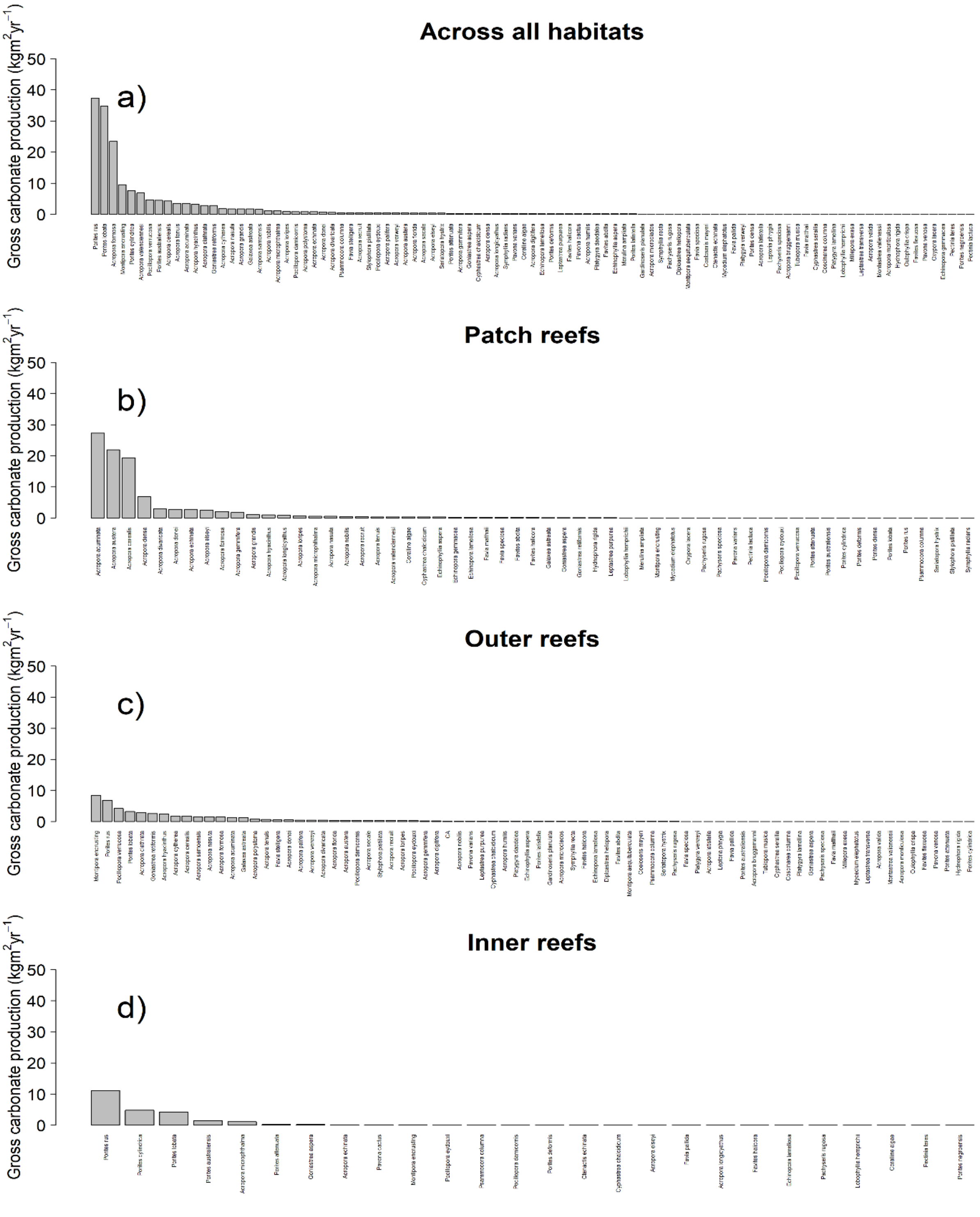

Figure B. Contribution of carbonate production (kg $CaCO_3$ m^{-2} y^{-1}) by coral species, from 24 sites in Palau, 2017. Panel **a)** shows the contribution of species across all 24 sites, panel **b)** shows the contribution of species across the 10 patch reef sites, panel **c)** shows the contribution of

species across the 8 outer reef sites and panel **d)** shows the contribution of species across the 6 inner reef sites.

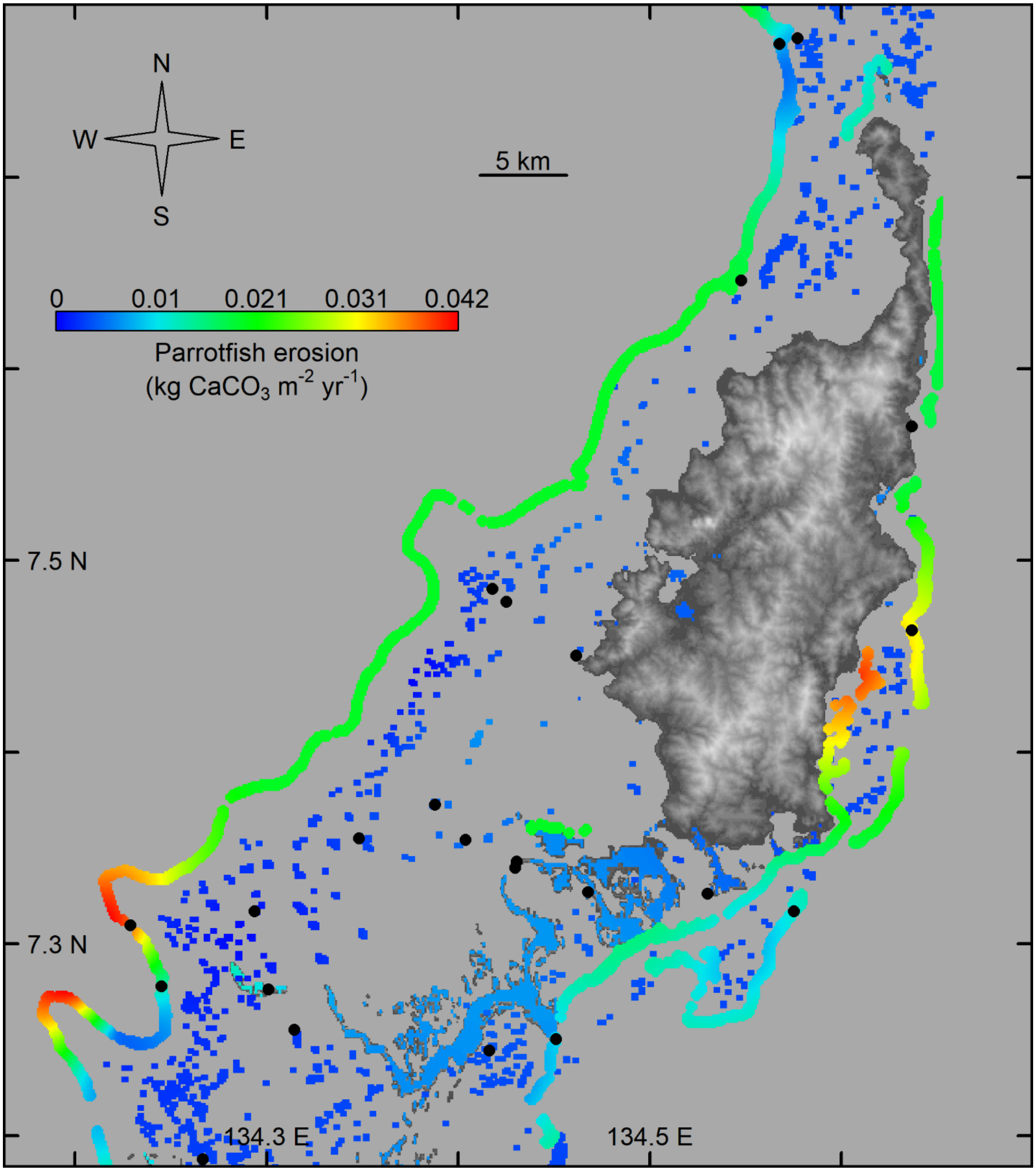

Figure C. Erosional estimates for parrotfishes (kg $CaCO_3$ m^{-2} yr^{-1}) using spatial kriging for shallow-water coral-reef habitats (including inner, outer, and patch reefs), from 24 sites in Palau, 2017. The kriged maps were generated in R (R Core Team, 2020) and the topography was generated using 'elevatr' in R (Hollister et al. 2020) using the source GL3 for

OpenTopography's data from the Shuttle Radar Topography Mission GL3,
https://portal.opentopography.org/raster?opentopoID=OTSRTM.042013.4326.1

Yap

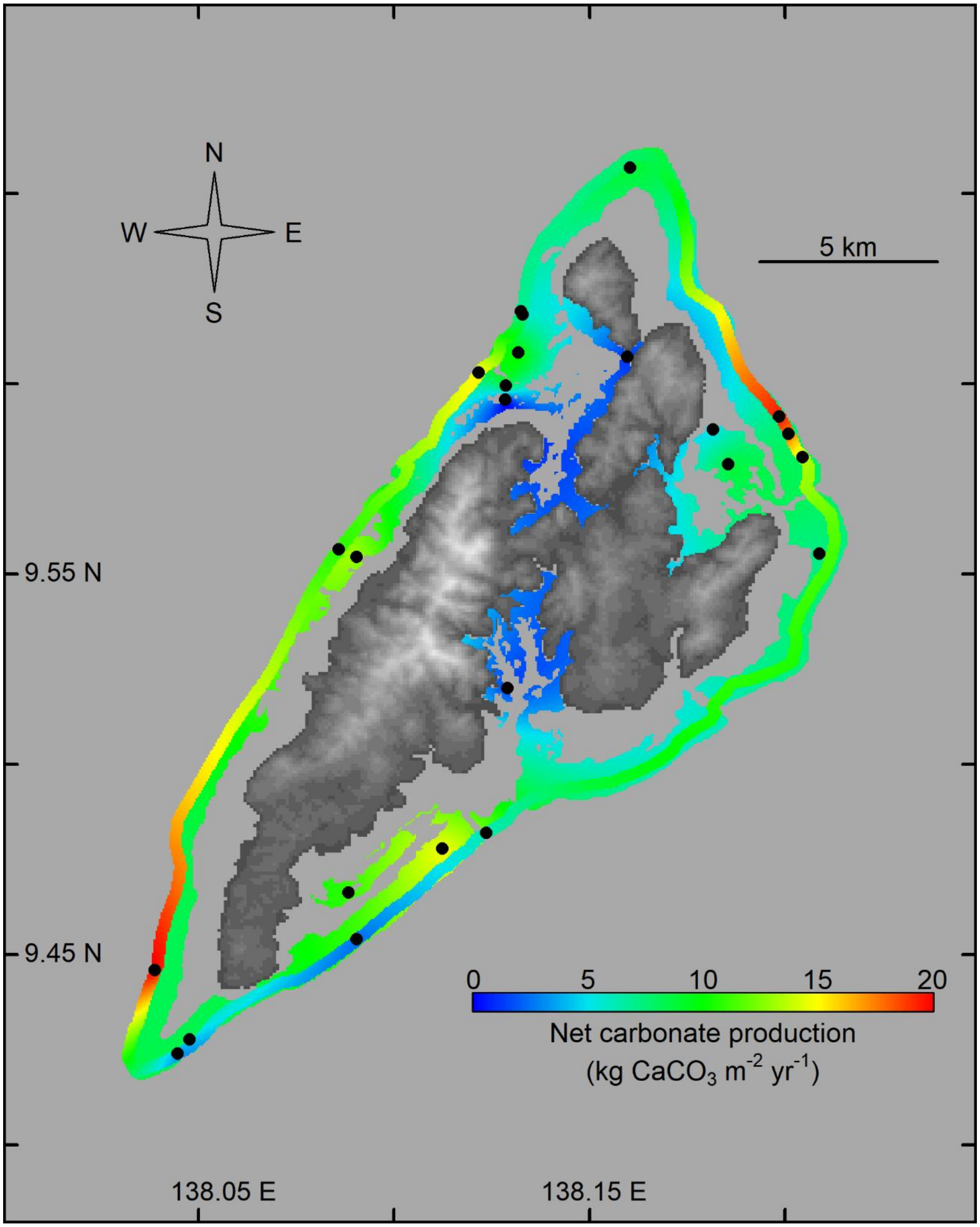

Figure D. Spatial kriging of the net shallow-water coral-reef carbonate production (kg $CaCO_3$ m^{-2} yr^{-1}) from 24 sites in Yap, 2017. The kriged maps were generated in R (R Core Team, 2020) and the topography was generated using ‘elevatr’ in R (Hollister et al. 2020) using the source GL3 for OpenTopography's data from the Shuttle Radar Topography Mission GL3, https://portal.opentopography.org/raster?opentopoID=OTSRTM.042013.4326.1

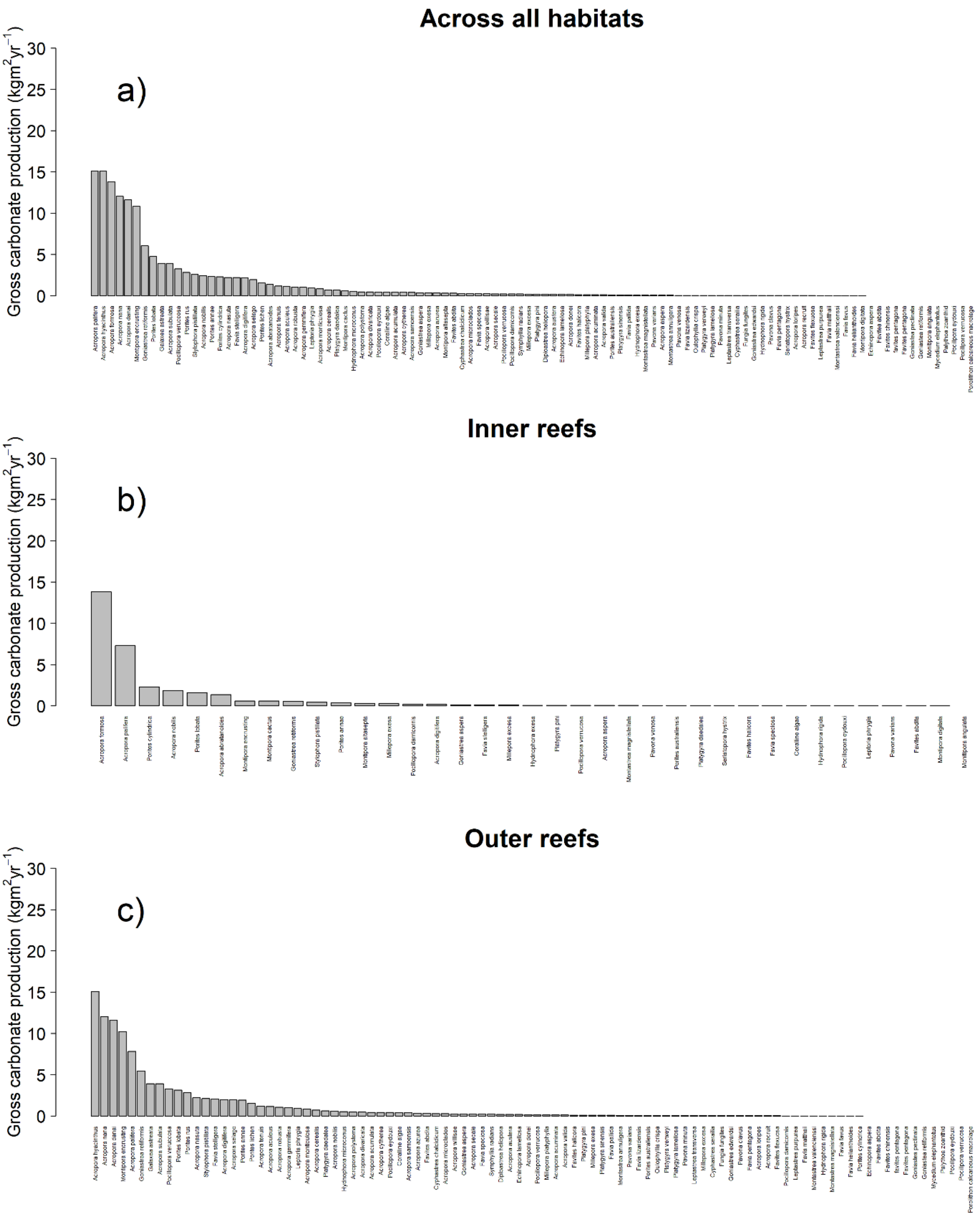

Figure E. Contribution of carbonate production (kg $CaCO_3$ m^{-2} y^{-1}) by coral species from 24 sites in Yap, 2017. Panel **a)** shows the contribution of species across all 24 sites, panel **b)** shows the contribution of species across the 14 inner reef sites, and panel **c)** shows the contribution of species across the 10 outer reef sites.

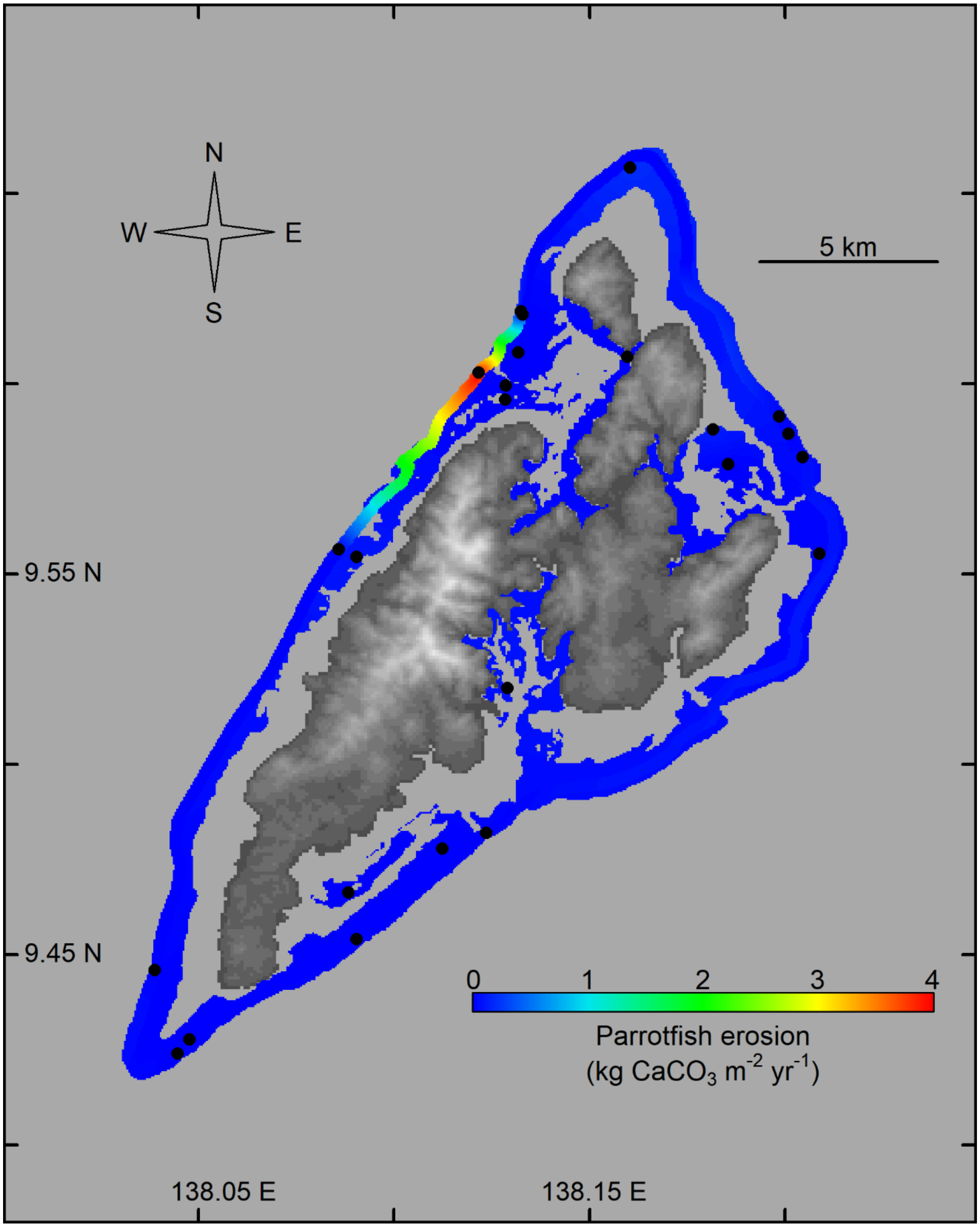

Figure F. Erosional estimates for parrotfishes (kg $CaCO_3$ m^{-2} yr^{-1}) using spatial kriging for shallow-water coral-reef habitats (including inner, outer, and patch reefs) from 24 sites in Yap, 2017. The kriged maps were generated in R (R Core Team, 2020) and the topography was generated using 'elevatr' in R (Hollister et al. 2020) using the source GL3 for OpenTopography's data from the Shuttle Radar Topography Mission GL3, https://portal.opentopography.org/raster?opentopoID=OTSRTM.042013.4326.1

Pohnpei

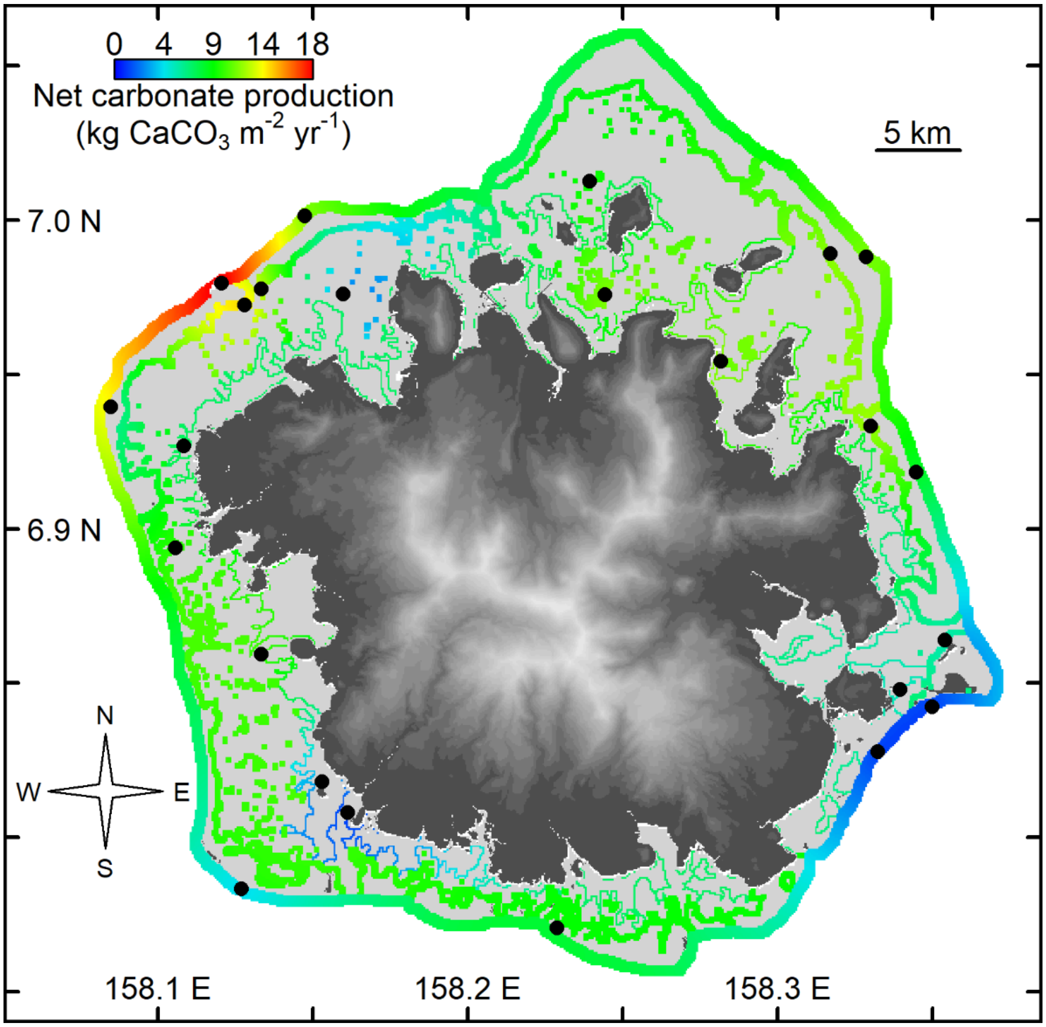

Figure G. Spatial kriging of the net shallow-water coral-reef carbonate production (kg $CaCO_3$ m^{-2} yr^{-1}) from 24 sites in Pohnpei, 2018. The kriged maps were generated in R (R Core Team, 2020) and the topography was generated using 'elevatr' in R (Hollister et al. 2020) using the source GL3 for OpenTopography's data from the Shuttle Radar Topography Mission GL3, https://portal.opentopography.org/raster?opentopoID=OTSRTM.042013.4326.1

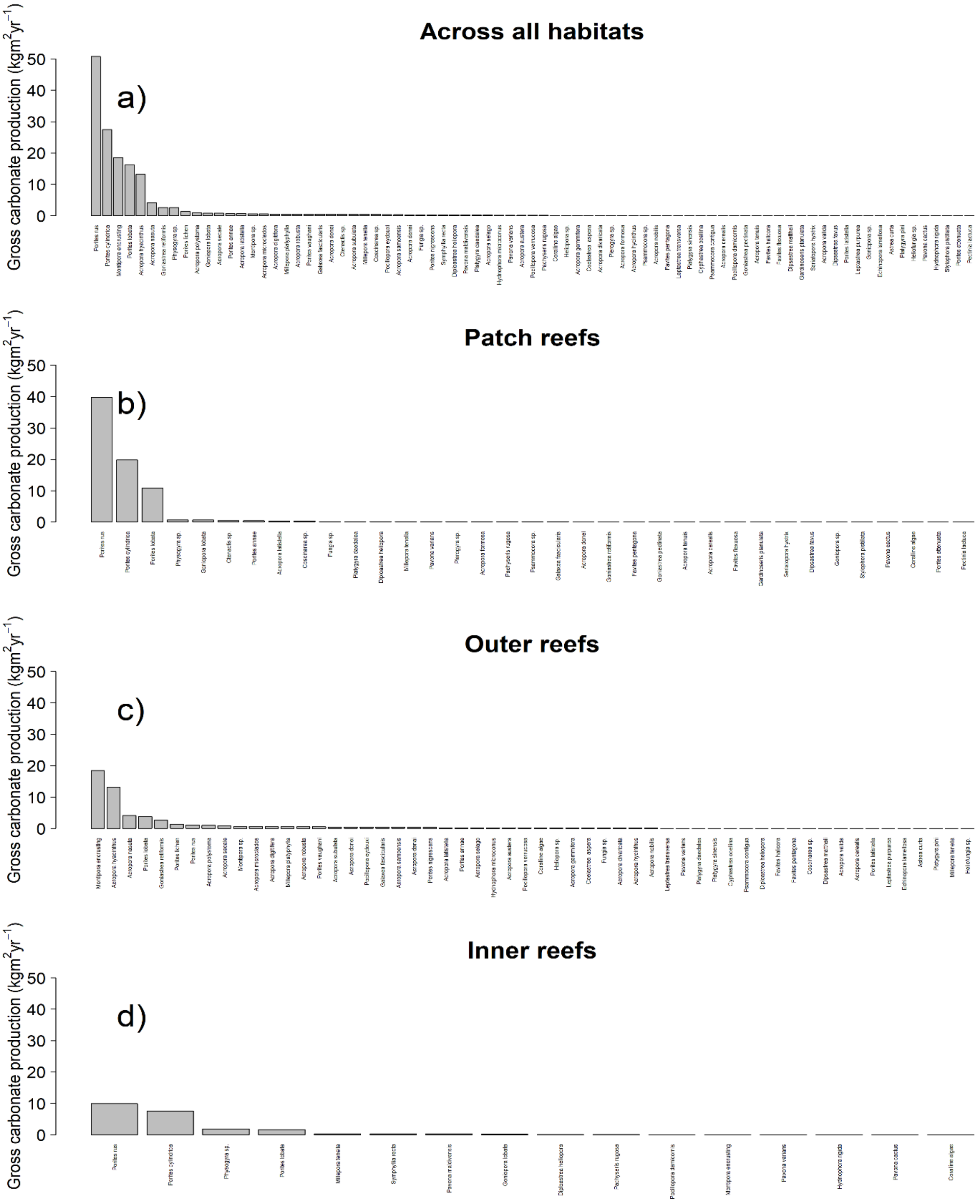

Figure H. Contribution of carbonate production (kg $CaCO_3$ m^{-2} y^{-1}) by coral species from 24 sites in Pohnpei, 2018. Panel **a)** shows the contribution of species across all 24 sites, panel **b)** shows the contribution of species across the 11 patch reef sites, panel **c)** shows the contribution of species across the 8 outer reef sites and panel **d)** shows the contribution of species across the 5 inner reef sites.

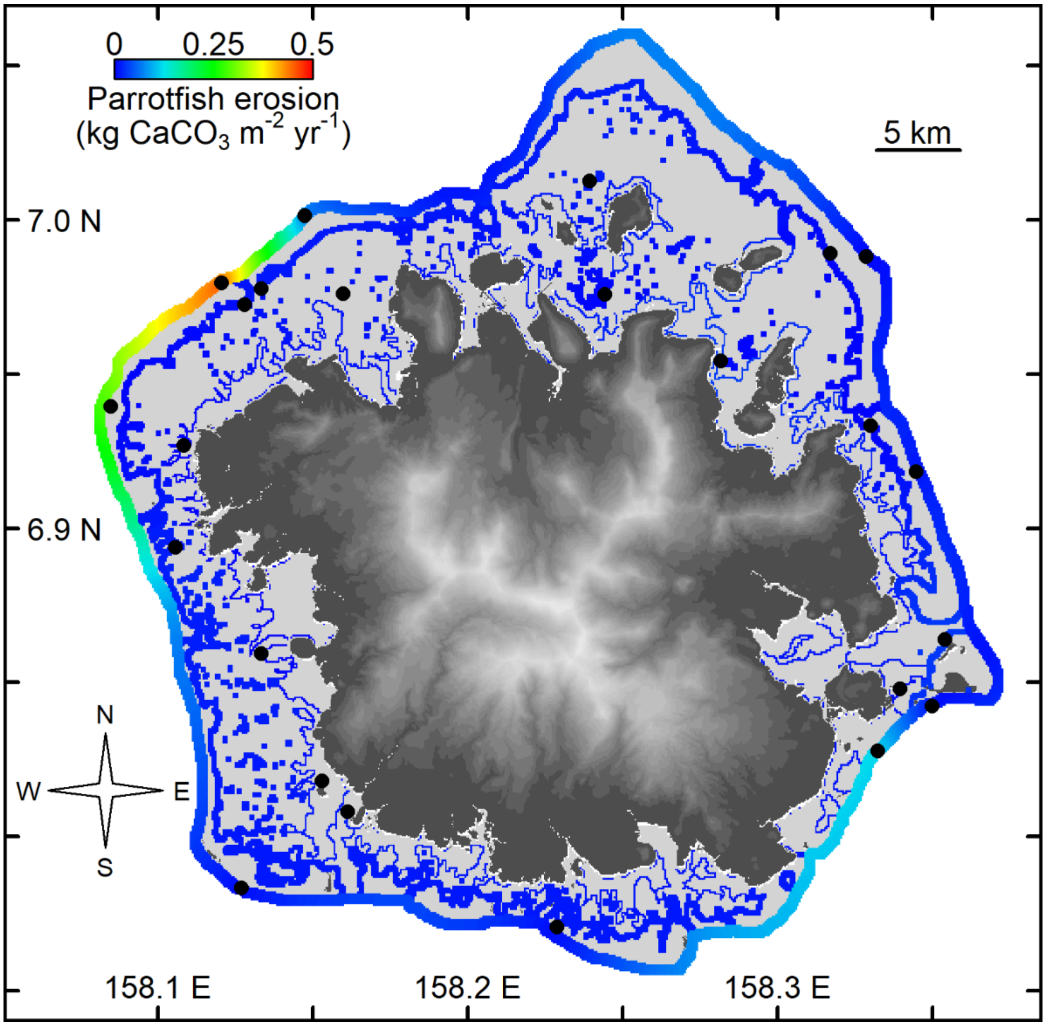

Figure I. Erosional estimates for parrotfishes (kg $CaCO_3$ m^{-2} yr^{-1}) using spatial kriging for shallow-water coral-reef habitats (including inner, outer, and patch reefs) from 24 sites in Pohnpei, 2018. The kriged maps were generated in R (R Core Team, 2020) and the topography was generated using 'elevatr' in R (Hollister et al. 2020) using the source GL3 for OpenTopography's data from the Shuttle Radar Topography Mission GL3, https://portal.opentopography.org/raster?opentopoID=OTSRTM.042013.4326.1

Kosrae

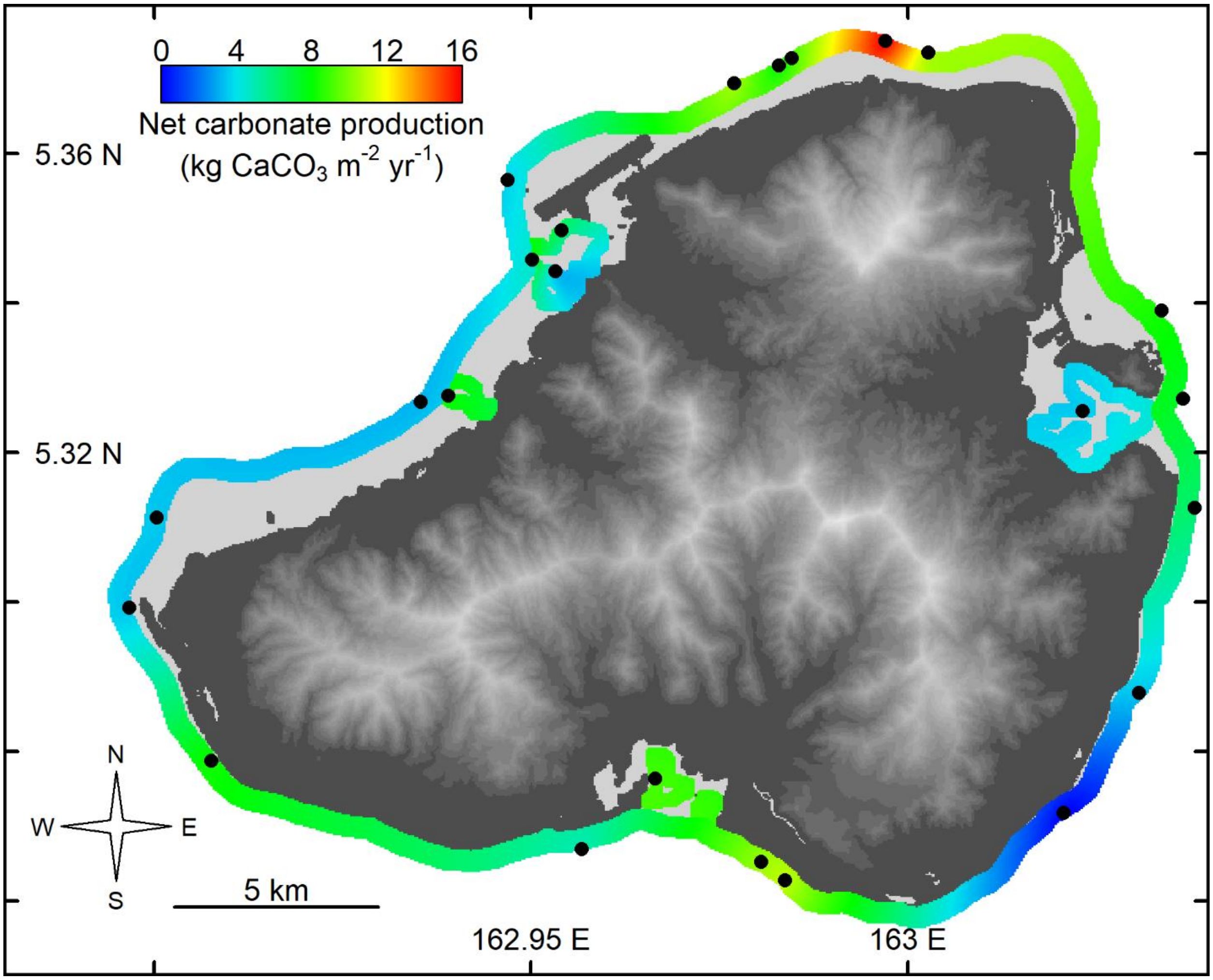

Figure J. Spatial kriging of the net shallow-water coral-reef carbonate production (kg $CaCO_3$ m^{-2} yr^{-1}) from 24 sites in Kosrae, 2018. The kriged maps were generated in R (R Core Team, 2020) and the topography was generated using 'elevatr' in R (Hollister et al. 2020) using the source GL3 for OpenTopography's data from the Shuttle Radar Topography Mission GL3, https://portal.opentopography.org/raster?opentopoID=OTSRTM.042013.4326.1

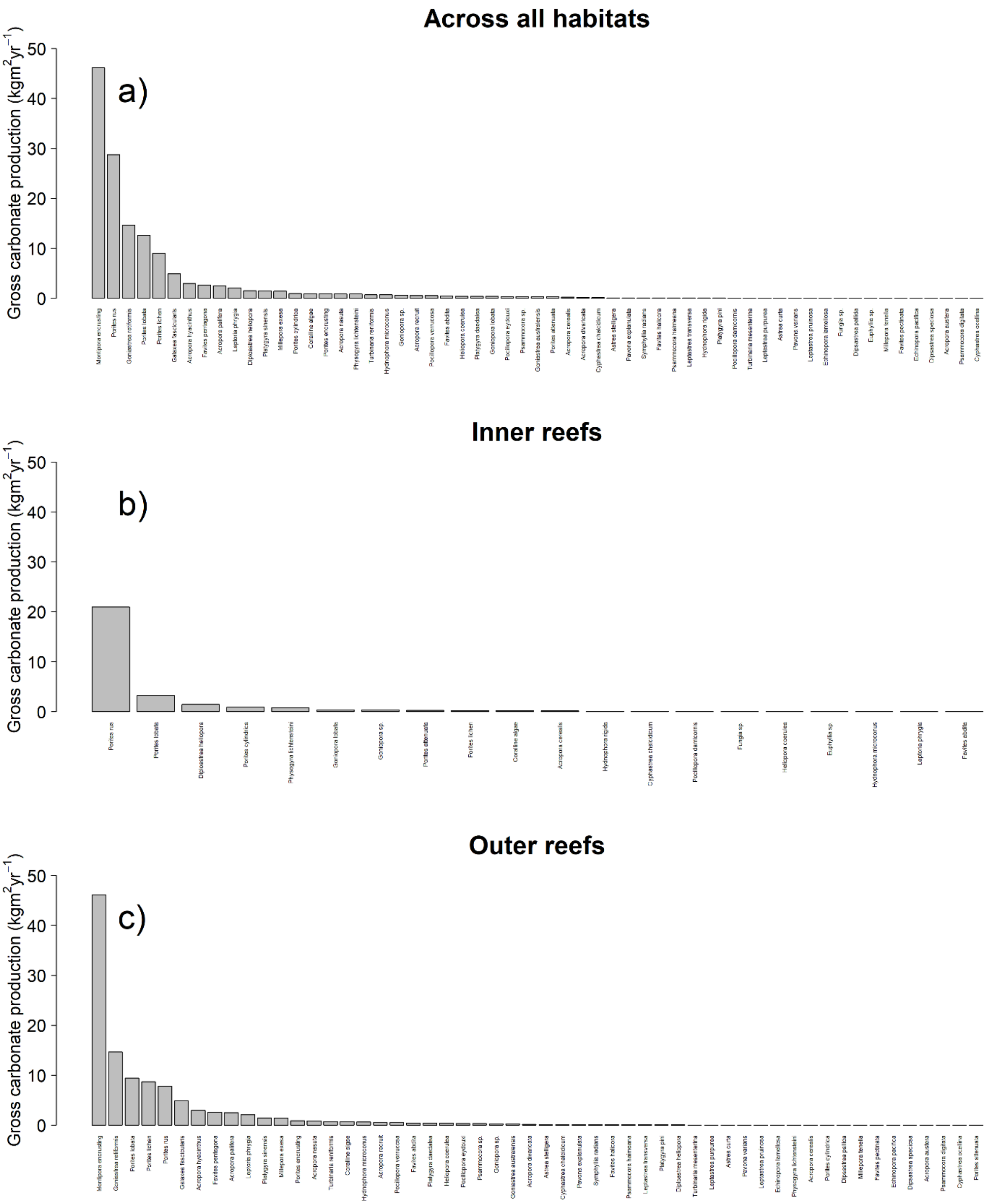

Figure K. Contribution of carbonate production (kg $CaCO_3$ m^{-2} y^{-1}) by coral species from 24 sites in Kosrae, 2018. Panel **a)** shows the contribution of species across all 24 sites, panel **b)** shows the contribution of species across the 6 inner reef sites, and panel **c)** shows the contribution of species across the 18 outer reef sites.

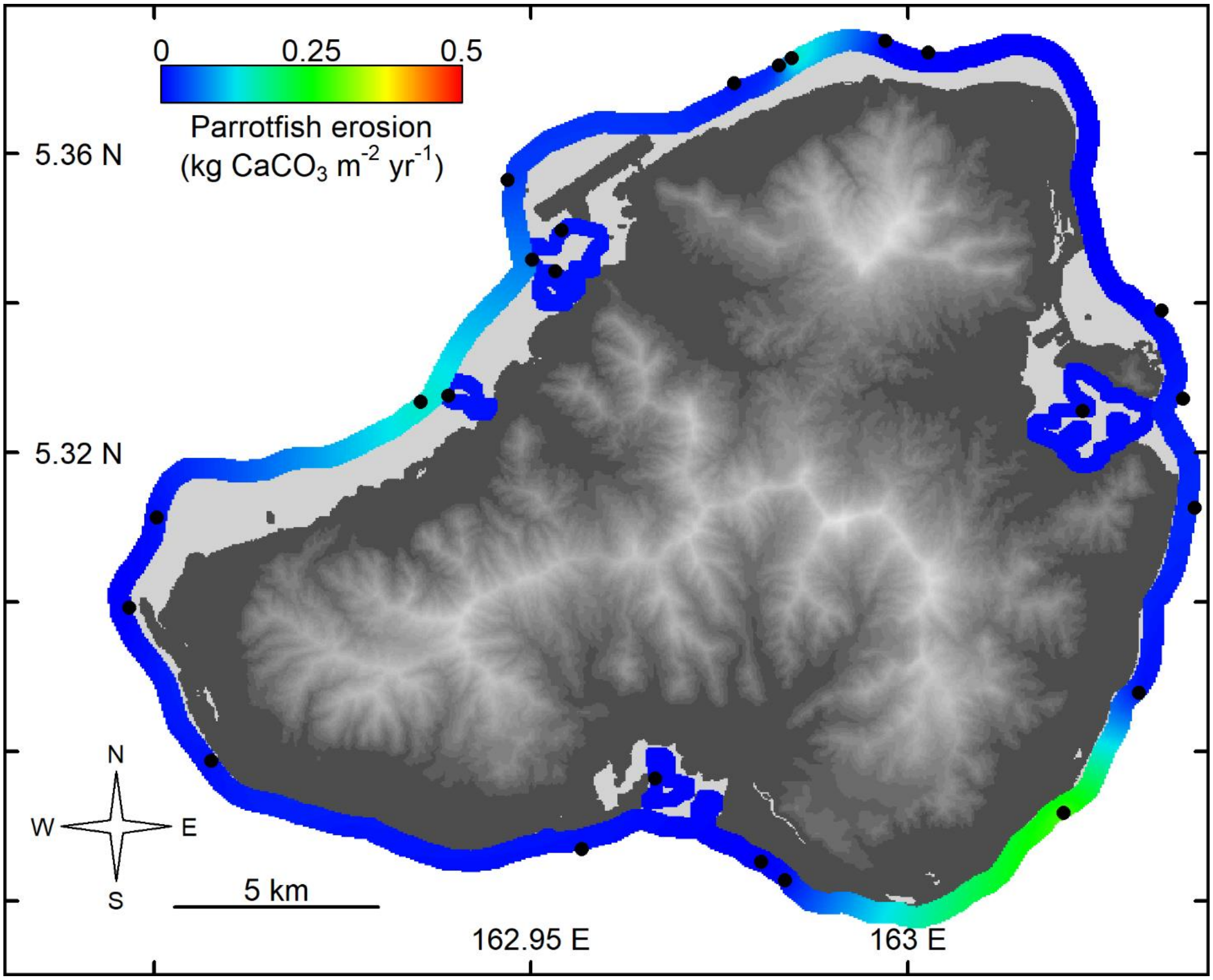

Figure L. Erosional estimates for parrotfishes (kg $CaCO_3$ m^{-2} yr^{-1}) using spatial kriging for shallow-water coral-reef habitats (including inner, outer, and patch reefs) from 24 sites in Kosrae, 2018. The kriged maps were generated in R (R Core Team, 2020) and the topography was generated using 'elevatr' in R (Hollister et al. 2020) using the source GL3 for OpenTopography's data from the Shuttle Radar Topography Mission GL3, https://portal.opentopography.org/raster?opentopoID=OTSRTM.042013.4326.1

Majuro

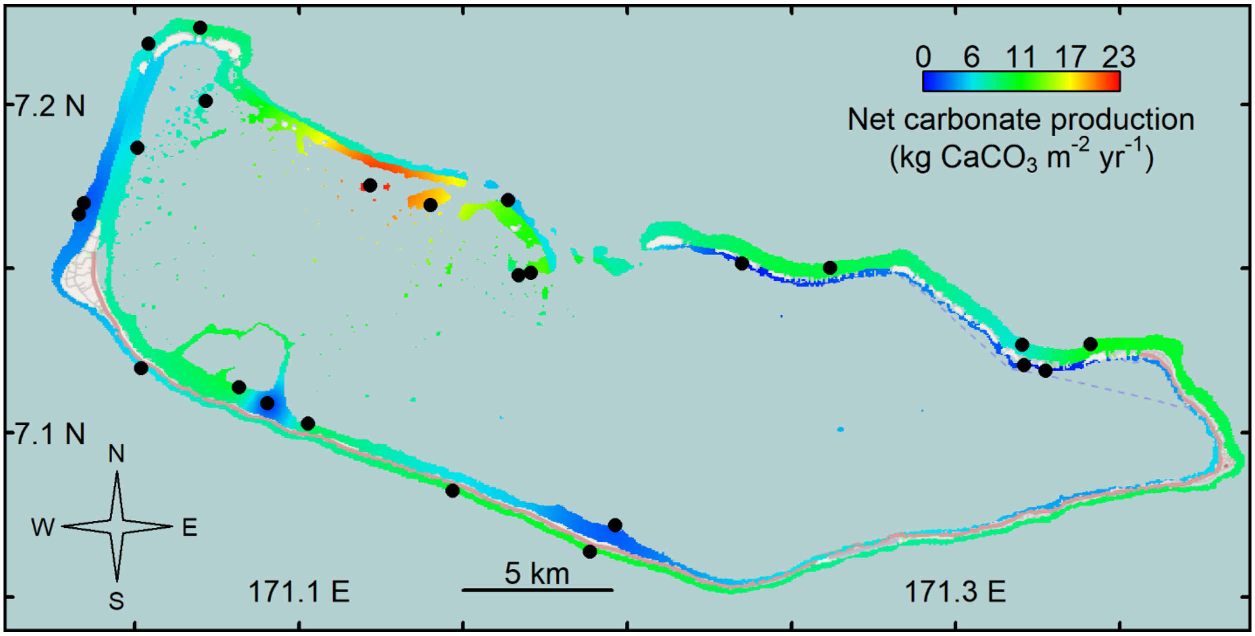

Figure M. Spatial kriging of the net shallow-water coral-reef carbonate production (kg $CaCO_3$ m^{-2} yr^{-1}) from 24 sites in Majuro, 2019. The outline of the atoll was derived from https://www.openstreetmap.org/. The maps were generated in R (R Core Team, 2020).

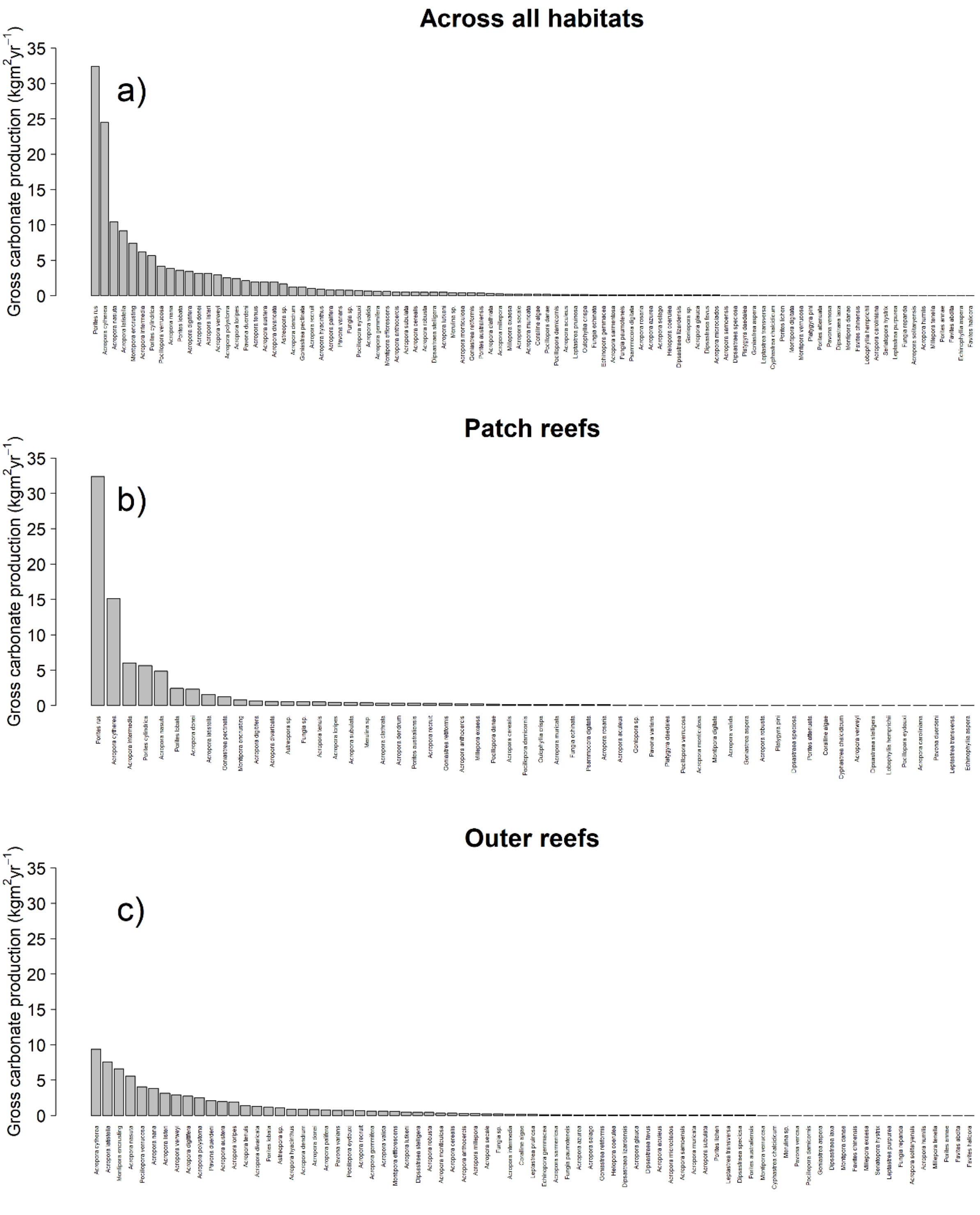

Figure N. Contribution of carbonate production (kg $CaCO_3$ m^{-2} y^{-1}) by coral species from 24 sites in Majuro, 2019. Panel **a)** shows the contribution of species across all 24 sites, panel **b)** shows the contribution of species across the 13 patch reef sites, and panel **c)** shows the contribution of species across the 11 outer reef sites.

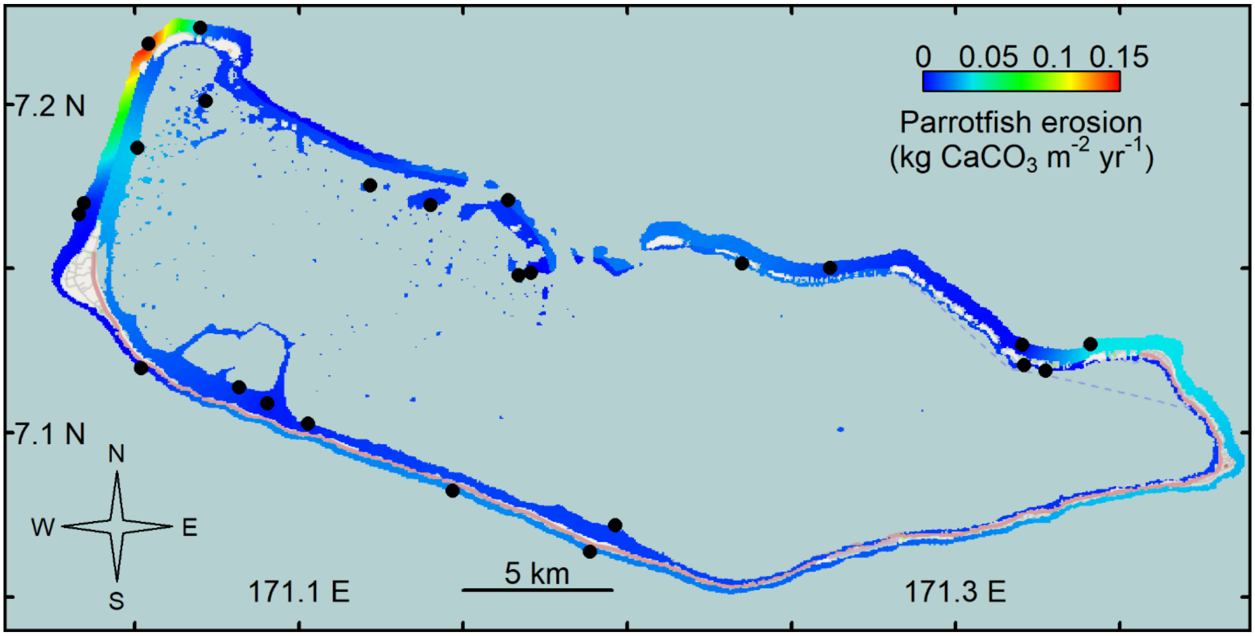

Figure O. Erosional estimates for parrotfishes (kg $CaCO_3$ m^{-2} yr^{-1}) using spatial kriging for shallow-water coral-reef habitats (including outer, and patch reefs) from 24 sites in Majuro, 2019. The outline of the atoll was derived from https://www.openstreetmap.org/. The maps were generated in R (R Core Team, 2020).

Kiritimati

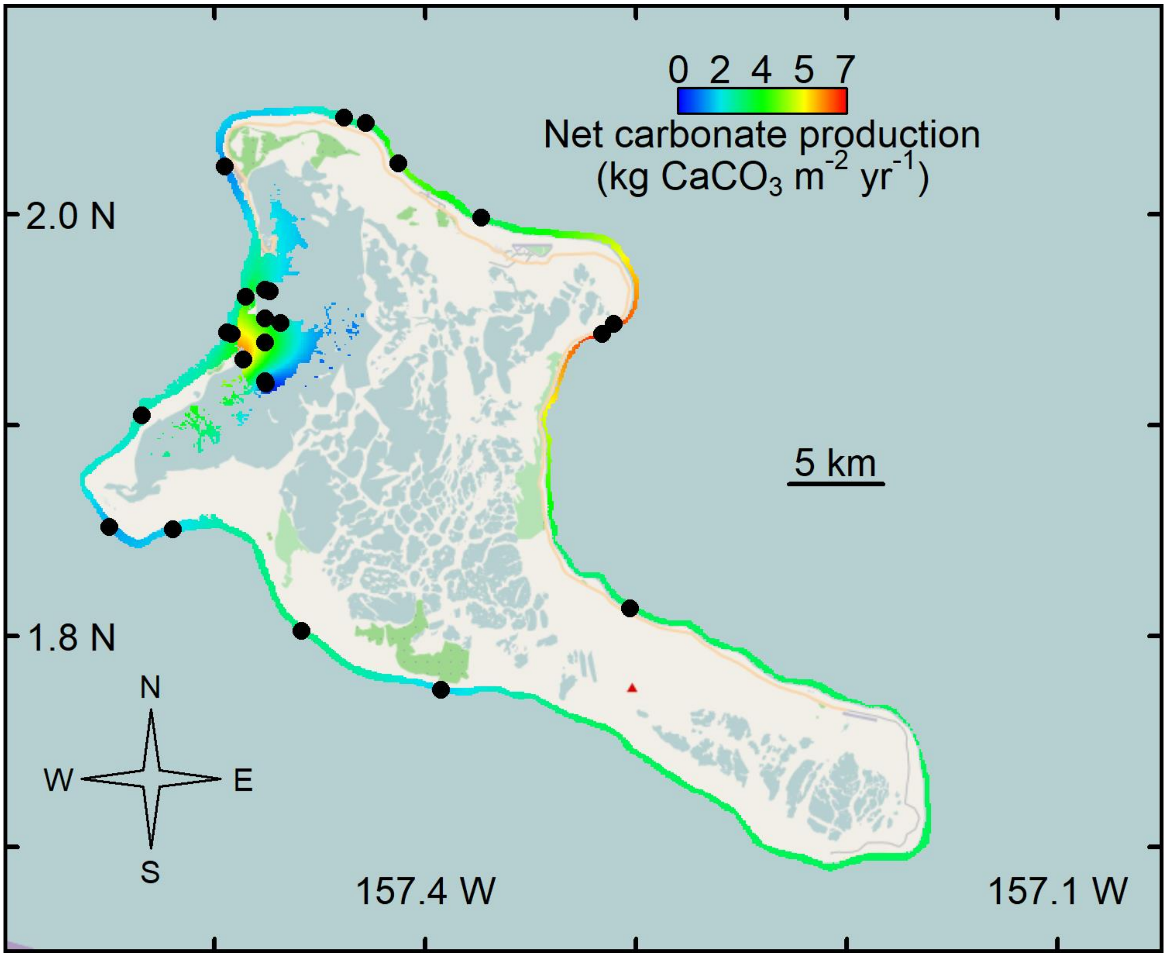

Figure P. Spatial kriging of the net shallow-water coral-reef carbonate production (kg $CaCO_3$ m^{-2} yr^{-1}) from 22 sites in Kiritimati, 2019. Note that the eastern reefs of Kiritimati were only lightly surveyed because of inclement ocean conditions. The outline and details of the atoll were derived from https://www.openstreetmap.org/. The maps were generated in R (R Core Team, 2020).

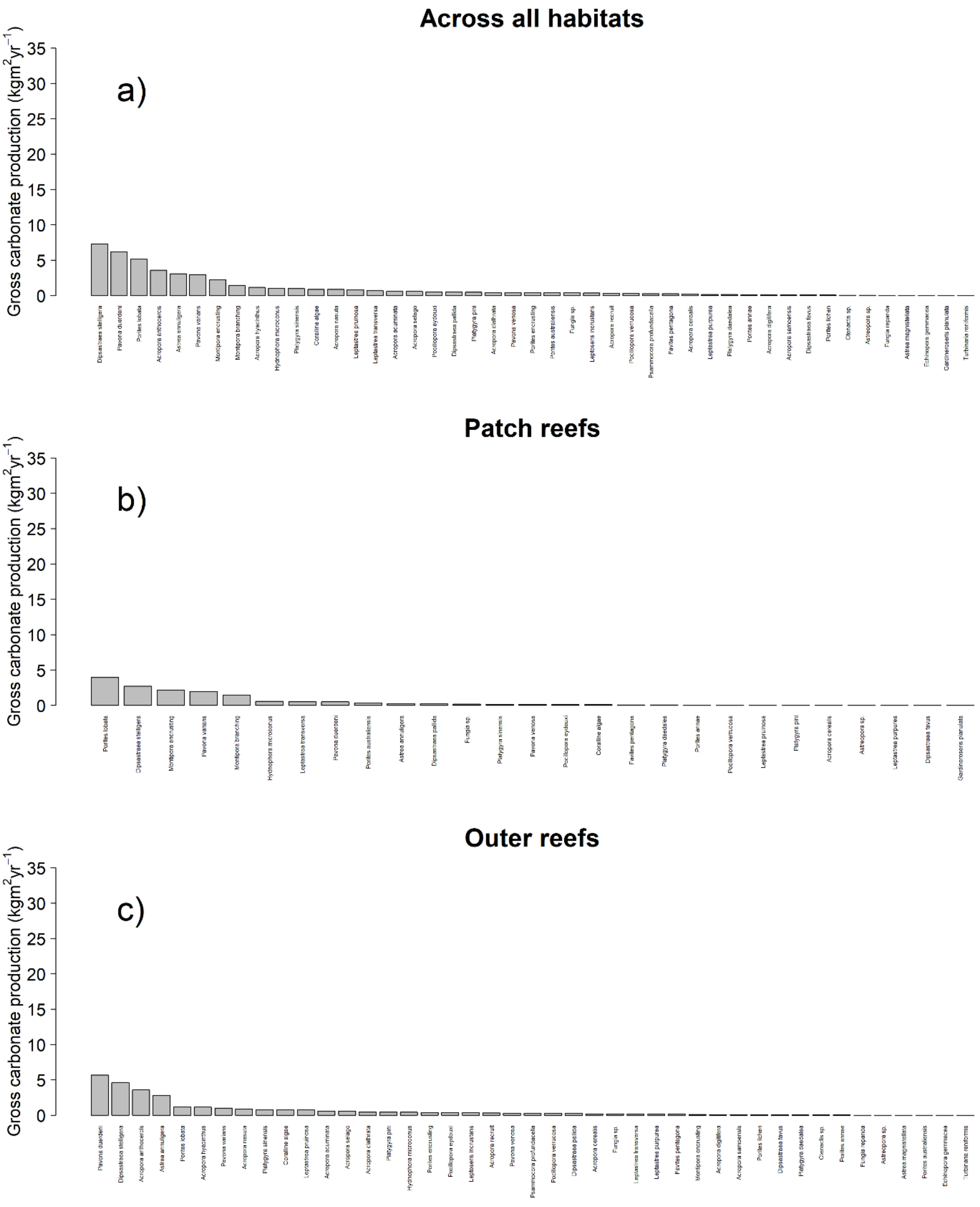

Figure Q. Contribution of carbonate production (kg $CaCO_3$ m^{-2} y^{-1}) by coral species from 22 sites in Kiritimati, 2019. Panel **a)** shows the contribution of species across all 22 sites, panel **b)** shows the contribution of species across the 8 patch reef sites, and panel **c)** shows the contribution of species across the 14 outer reef sites.

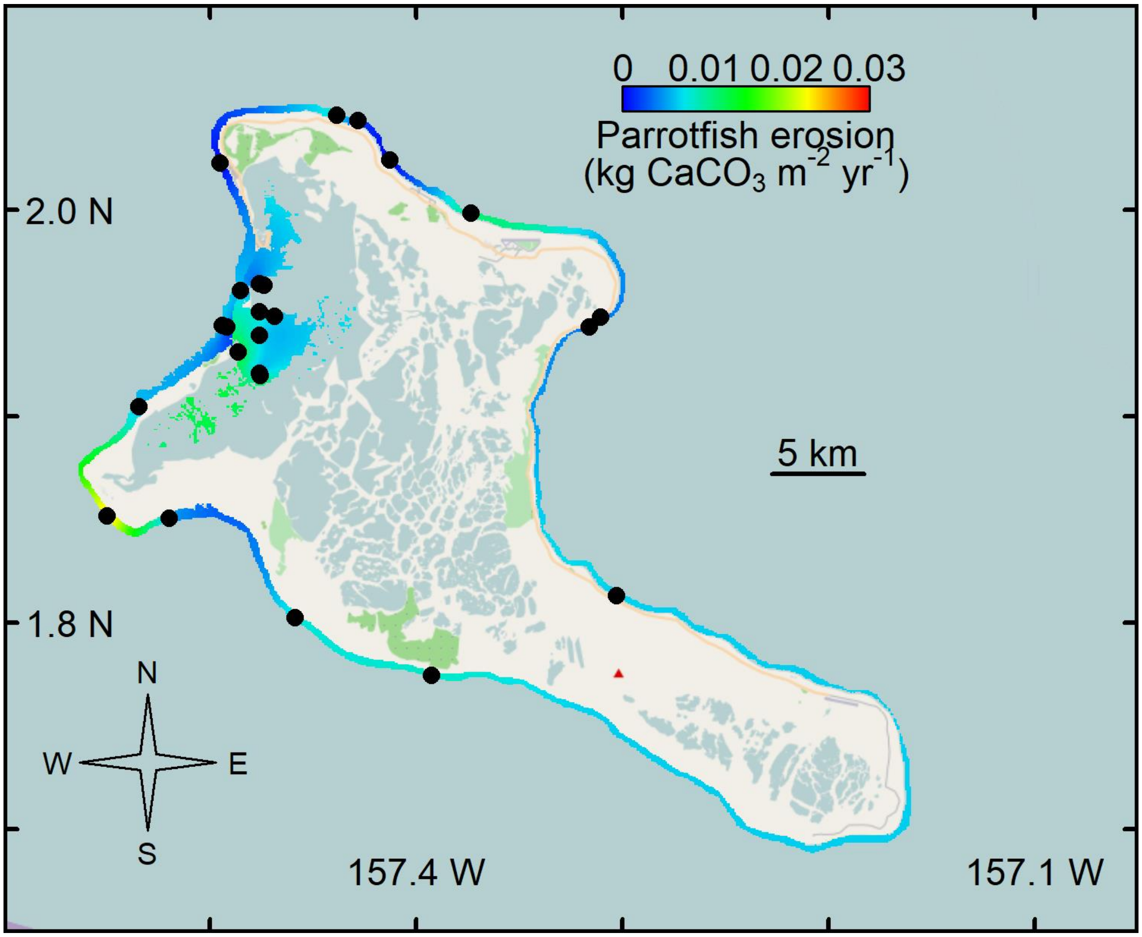

Figure R. Erosional estimates for parrotfishes (kg $CaCO_3$ m^{-2} yr^{-1}) using spatial kriging for shallow-water coral-reef habitats (including inner, outer, and patch reefs) from 22 sites in Kiritimati, 2019. Note that the eastern reefs of Kiritimati were only lightly surveyed because of inclement ocean conditions. The outline and details of the atoll were derived from https://www.openstreetmap.org/. The maps were generated in R (R Core Team, 2020).

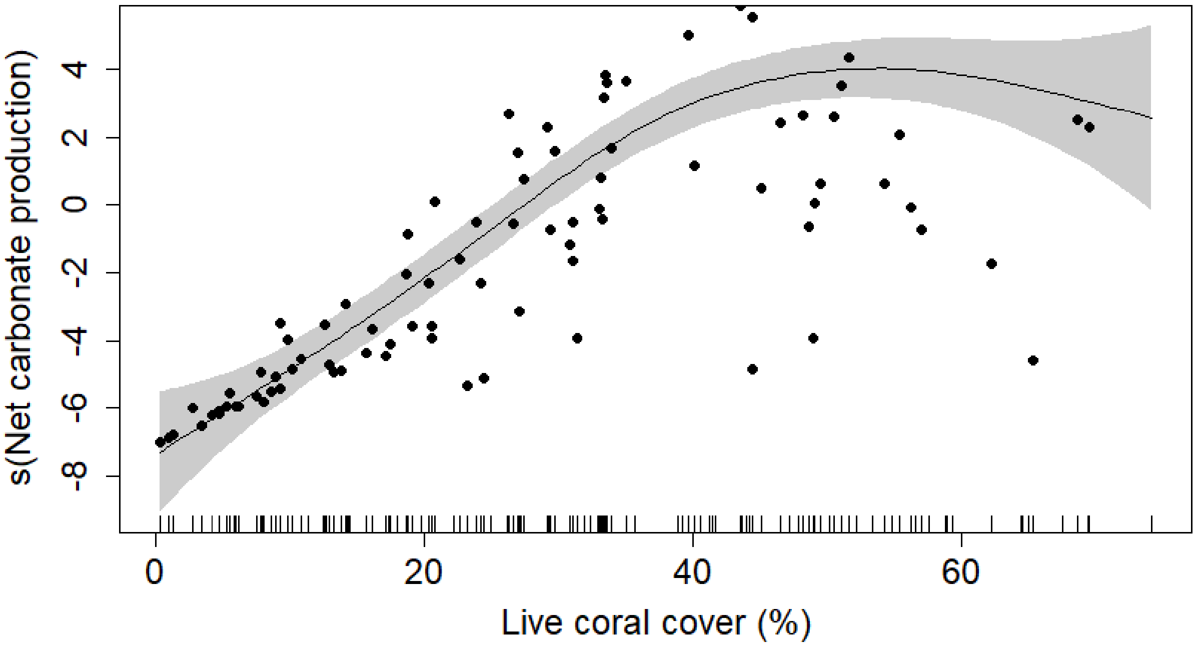

Figure S. Generalized additive model examining the relationship between live coral cover and net carbonate production at all 142 sites across the western and central tropical Pacific Ocean. Where the shading represents the 95% confidence intervals, s represents the smoothing function, and the rug plots represent the raw data. Twenty-four of the 142 sites were located in the western Pacific Ocean at each of Palau and Yap in 2017, 24 sites were located progressively further east at each of Pohnpei and Kosrae in 2018, and at Majuro in 2019, and 22 sites were located in the central Pacific Ocean at Kiritimati in 2019.

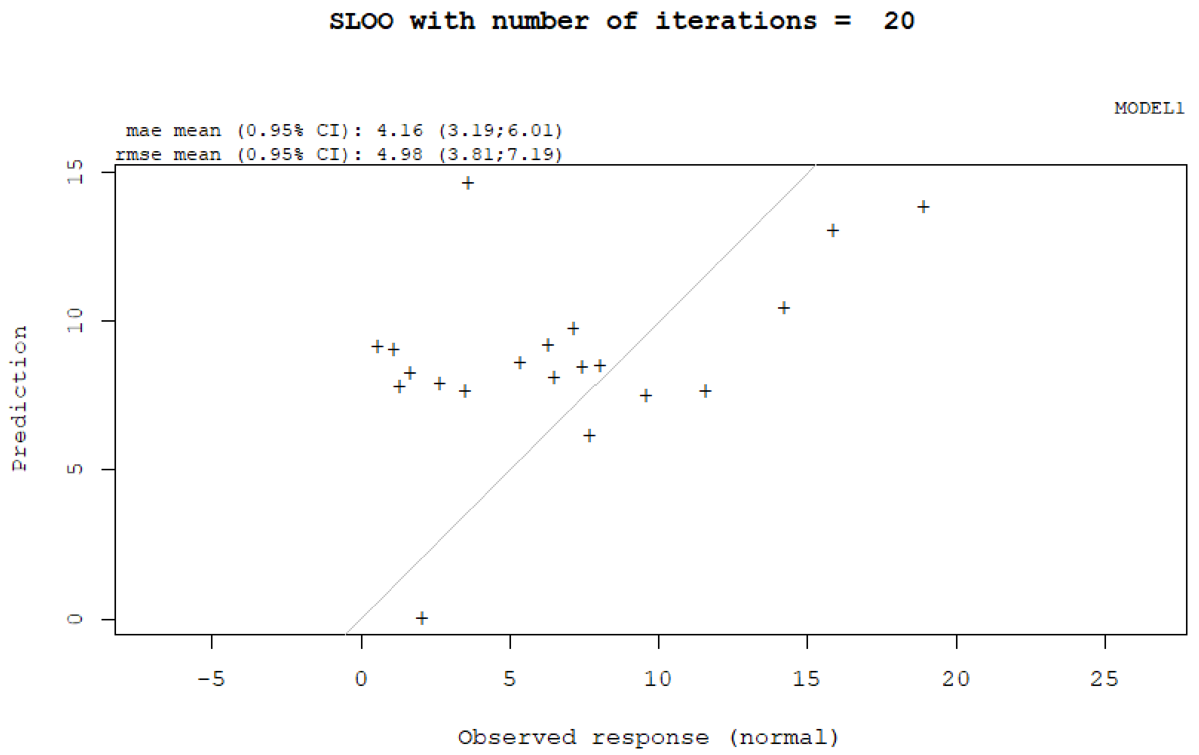

Figure T. Spatial cross-validation using spatial leave-one-out cross validation, comparing the observed response of carbonate production against the predicted response of carbonate production using INLAutils (Lucas et al. 2020) for all 142 sites across the western and central tropical Pacific Ocean, and where the identity function (i.e., y=x) is depicted by the line.

Table 1: Site locations and corresponding model output mean and standard deviation (SD) including Live Coral Cover (LCC), gross production, net production, bioerosion and rugosity.

Island	Site	Reef location	Mean LCC	SD LCC	Latitude	Longitude	Mean gross production	SD gross production	Mean net production	SD net production	Mean bioerosion	SD bioerosion	Mean rugosity	SD rugosity
Kiritimati	1	outer	61.667	25.727	1.852	-157.550	1.149	0.513	1.161	0.518	0.045	0.018	1.083	0.055
Kiritimati	2	outer	80.333	51.087	1.944	-157.494	1.274	0.700	1.299	0.686	0.032	0.030	1.131	0.084
Kiritimati	3	outer	126.500	19.066	1.905	-157.535	1.895	0.624	1.931	0.622	0.021	0.011	1.188	0.130
Kiritimati	4	outer	197.333	126.168	1.944	-157.316	6.637	4.692	6.681	4.691	0.014	0.004	1.057	0.088
Kiritimati	5	outer	101.667	57.933	2.046	-157.439	2.194	1.196	2.233	1.205	0.018	0.020	1.149	0.076
Kiritimati	6	outer	144.500	60.596	1.999	-157.374	2.802	1.312	2.843	1.310	0.017	0.008	1.098	0.045
Kiritimati	7	outer	79.500	49.581	1.775	-157.393	1.280	0.860	1.322	0.861	0.015	0.009	1.058	0.046
Kiritimati	8	outer	74.833	39.872	1.851	-157.520	1.429	0.894	1.467	0.893	0.019	0.013	1.071	0.038
Kiritimati	9	outer	171.667	90.266	2.043	-157.428	2.623	1.890	2.650	1.889	0.030	0.006	1.139	0.062
Kiritimati	10	outer	113.667	40.727	1.803	-157.459	2.631	1.133	2.674	1.137	0.015	0.009	1.179	0.016
Kiritimati	11	outer	47.333	41.157	2.023	-157.495	0.990	0.999	1.031	0.998	0.016	0.008	1.236	0.098
Kiritimati	12	outer	179.500	73.571	2.025	-157.413	4.008	2.641	4.047	2.635	0.018	0.015	1.110	0.043
Kiritimati	13	outer	161.333	55.186	1.944	-157.492	3.393	0.893	3.446	0.894	0.004	0.003	1.315	0.051
Kiritimati	14	outer	129.000	55.674	1.961	-157.485	2.337	1.303	2.386	1.306	0.009	0.009	1.243	0.135
Kiritimati	15	patch	9.833	11.940	1.921	-157.476	0.169	0.190	0.226	0.190	0.000	0.000	1.015	0.009
Kiritimati	16	patch	297.500	88.652	1.940	-157.476	4.093	1.476	4.140	1.473	0.010	0.007	1.158	0.129
Kiritimati	17	patch	92.833	38.768	1.964	-157.474	1.639	0.572	1.693	0.574	0.003	0.004	1.138	0.048
Kiritimati	18	patch	143.000	27.210	1.965	-157.476	1.998	0.432	2.048	0.432	0.008	0.003	1.107	0.071
Kiritimati	19	patch	174.500	77.400	1.951	-157.476	2.954	1.755	3.002	1.759	0.009	0.004	1.230	0.150
Kiritimati	20	patch	414.333	77.397	1.931	-157.487	6.457	2.259	6.507	2.256	0.007	0.005	1.216	0.049
Kiritimati	21	patch	132.500	43.666	1.949	-157.469	2.118	0.940	2.171	0.940	0.004	0.002	1.248	0.148
Kiritimati	22	patch	3.167	5.154	1.920	-157.476	0.036	0.059	0.093	0.059	0.000	0.000	1.010	0.009
Kosrae	1	outer	222.167	149.839	5.267	162.957	5.045	2.822	5.070	2.823	0.013	0.004	1.184	0.115
Kosrae	2	outer	436.667	126.410	5.279	162.908	9.117	3.145	9.141	3.146	0.013	0.008	1.240	0.135
Kosrae	3	outer	292.667	78.270	5.265	162.981	10.859	4.583	10.892	4.581	0.004	0.005	1.378	0.215
Kosrae	4	outer	357.000	89.170	5.373	163.003	9.990	2.795	9.905	2.813	0.123	0.149	1.142	0.067
Kosrae	5	outer	478.333	141.020	5.375	162.997	16.415	5.460	16.432	5.450	0.021	0.041	1.221	0.099
Kosrae	6	outer	271.000	85.769	5.299	162.897	3.910	2.980	3.940	2.983	0.007	0.009	1.328	0.099
Kosrae	7	outer	205.000	37.170	5.327	162.935	3.152	1.197	3.174	1.197	0.015	0.011	1.161	0.030
Kosrae	8	outer	238.500	63.667	5.313	163.038	6.537	1.419	6.574	1.418	0.001	0.001	1.046	0.025
Kosrae	9	outer	274.000	76.780	5.327	163.036	7.839	2.170	7.871	2.168	0.004	0.003	1.064	0.027
Kosrae	10	outer	205.500	76.673	5.356	162.947	3.955	2.034	3.532	1.706	0.460	0.946	1.458	0.147
Kosrae	11	outer	231.500	179.679	5.288	163.031	1.749	1.815	1.768	1.801	0.018	0.024	1.083	0.043
Kosrae	12	outer	47.833	72.544	5.272	163.021	0.902	1.510	0.935	1.504	0.004	0.006	1.091	0.095
Kosrae	13	outer	310.500	63.570	5.372	162.983	6.606	2.391	6.596	2.456	0.047	0.097	1.186	0.132
Kosrae	14	outer	269.500	20.888	5.339	163.034	8.637	0.468	8.671	0.466	0.003	0.003	1.146	0.032
Kosrae	15	outer	486.000	128.735	5.263	162.984	11.555	5.043	11.577	5.041	0.016	0.022	1.451	0.251
Kosrae	16	outer	336.000	58.839	5.369	162.977	10.692	2.277	10.722	2.279	0.007	0.007	1.103	0.016

Kosrae	17	outer	314.000	60.992	5.311	162.900	3.256	1.538	3.156	1.459	0.137	0.216	1.308	0.075
Kosrae	18	outer	339.000	80.880	5.373	162.985	8.764	2.025	8.780	2.029	0.022	0.016	1.202	0.060
Kosrae	19	inner	203.667	226.681	5.326	163.023	4.776	4.951	4.812	4.951	0.001	0.004	1.229	0.140
Kosrae	20	inner	190.667	93.234	5.344	162.953	3.492	1.707	3.523	1.707	0.006	0.003	1.198	0.129
Kosrae	21	inner	450.500	140.891	5.328	162.939	7.558	4.699	7.589	4.703	0.007	0.007	1.458	0.279
Kosrae	22	inner	401.333	171.907	5.346	162.950	8.254	3.578	8.277	3.584	0.014	0.012	1.423	0.121
Kosrae	23	inner	510.000	187.553	5.276	162.966	10.589	6.303	10.617	6.300	0.009	0.015	1.583	0.363
Kosrae	24	inner	293.500	104.313	5.350	162.954	6.339	2.966	6.371	2.964	0.005	0.006	1.609	0.309
Majuro	1	outer	335.000	25.768	7.125	171.262	10.859	2.914	10.913	2.915	0.012	0.007	1.146	0.192
Majuro	2	outer	329.000	90.830	7.223	171.070	9.501	2.535	9.566	2.535	0.001	0.001	1.005	0.013
Majuro	3	outer	172.833	109.788	7.167	171.033	4.206	2.926	4.248	2.930	0.023	0.013	1.170	0.101
Majuro	4	outer	108.167	31.096	7.170	171.034	2.525	1.082	2.567	1.073	0.024	0.019	1.209	0.078
Majuro	5	outer	187.500	55.544	7.120	171.052	6.202	2.472	6.260	2.471	0.007	0.005	1.116	0.059
Majuro	6	outer	187.000	87.489	7.171	171.164	4.994	1.225	5.043	1.227	0.016	0.021	1.125	0.071
Majuro	7	outer	411.667	70.441	7.127	171.341	11.916	1.933	11.970	1.929	0.011	0.010	1.019	0.010
Majuro	8	outer	207.500	63.689	7.082	171.147	7.146	2.239	7.195	2.235	0.017	0.016	1.148	0.086
Majuro	9	outer	249.667	94.629	7.064	171.189	8.255	3.343	8.286	3.345	0.034	0.052	1.081	0.044
Majuro	10	outer	296.667	45.946	7.219	171.054	8.796	3.527	8.716	3.655	0.145	0.276	1.159	0.099
Majuro	11	outer	261.333	106.680	7.127	171.320	7.395	3.143	7.446	3.139	0.014	0.019	1.019	0.004
Majuro	12	patch	266.333	176.240	7.148	171.167	6.510	4.671	6.559	4.665	0.016	0.010	1.274	0.063
Majuro	13	patch	291.167	179.204	7.187	171.051	5.732	2.732	5.777	2.736	0.021	0.024	1.217	0.131
Majuro	14	patch	542.500	178.947	7.103	171.103	7.663	2.739	7.725	2.740	0.003	0.003	1.220	0.087
Majuro	15	patch	13.333	17.061	7.119	171.327	0.274	0.329	0.339	0.329	0.000	0.001	1.005	0.009
Majuro	16	patch	442.167	260.901	7.169	171.140	12.742	7.195	12.799	7.197	0.008	0.005	1.257	0.139
Majuro	17	patch	588.833	232.765	7.114	171.082	9.401	5.106	9.465	5.108	0.002	0.002	1.194	0.108
Majuro	18	patch	350.000	152.493	7.175	171.122	15.132	5.889	15.185	5.891	0.012	0.007	1.265	0.100
Majuro	19	patch	78.833	34.505	7.072	171.196	2.122	0.958	2.161	0.948	0.026	0.031	1.167	0.115
Majuro	20	patch	52.500	60.411	7.109	171.090	1.095	1.403	1.157	1.403	0.003	0.002	1.086	0.027
Majuro	21	patch	86.500	70.415	7.121	171.321	1.522	1.267	1.581	1.259	0.006	0.013	1.014	0.005
Majuro	22	patch	645.000	173.623	7.149	171.171	13.519	3.052	13.576	3.052	0.008	0.003	1.247	0.088
Majuro	23	patch	388.333	142.315	7.201	171.072	6.285	2.710	6.319	2.699	0.031	0.034	1.098	0.099
Majuro	24	patch	34.500	33.662	7.152	171.235	0.532	0.516	0.593	0.519	0.005	0.007	1.040	0.004
Palau	1	outer	142.667	61.805	7.770	134.568	4.164	1.906	4.189	1.898	0.032	0.060	1.098	0.060
Palau	2	outer	439.167	64.322	7.773	134.577	13.673	2.751	13.724	2.751	0.006	0.006	1.135	0.038
Palau	3	outer	92.167	67.113	7.250	134.451	3.572	2.878	3.622	2.879	0.006	0.004	1.271	0.091
Palau	4	outer	54.667	41.409	7.317	134.575	1.504	1.088	1.555	1.086	0.007	0.011	1.000	0.000
Palau	5	outer	644.500	89.440	7.310	134.229	15.869	2.141	15.880	2.117	0.046	0.051	1.179	0.082
Palau	6	outer	89.500	46.642	7.464	134.637	2.022	0.823	2.040	0.763	0.039	0.067	1.000	0.000
Palau	7	outer	98.167	66.004	7.570	134.637	3.072	2.092	3.107	2.089	0.022	0.032	1.088	0.062
Palau	8	outer	575.333	145.642	7.278	134.245	17.377	7.374	17.428	7.374	0.006	0.010	1.174	0.057
Palau	9	inner	330.667	92.865	7.276	134.301	6.972	3.435	7.008	3.443	0.021	0.030	1.324	0.169

Palau	10	inner	622.333	51.399	7.450	134.461	5.293	2.795	5.348	2.795	0.003	0.002	1.180	0.038
Palau	11	inner	243.833	148.742	7.326	134.530	1.941	1.092	1.997	1.092	0.001	0.001	1.283	0.120
Palau	12	inner	562.333	117.595	7.327	134.468	6.977	4.171	7.029	4.171	0.005	0.003	1.558	0.169
Palau	13	inner	416.333	87.744	7.343	134.431	5.934	3.195	5.990	3.195	0.001	0.001	1.372	0.117
Palau	14	inner	486.000	161.789	7.340	134.430	6.417	4.585	6.469	4.589	0.005	0.007	1.256	0.092
Palau	15	patch	501.833	169.899	7.646	134.548	14.075	5.011	14.127	5.010	0.005	0.004	1.092	0.112
Palau	16	patch	533.833	210.469	7.355	134.348	8.769	3.970	8.824	3.970	0.002	0.002	1.143	0.095
Palau	17	patch	593.000	124.284	7.485	134.418	14.183	2.402	14.238	2.401	0.002	0.002	1.389	0.151
Palau	18	patch	569.833	135.152	7.354	134.404	6.313	3.852	6.361	3.855	0.008	0.006	1.070	0.055
Palau	19	patch	481.333	156.376	7.317	134.294	9.714	3.059	9.765	3.057	0.006	0.006	1.205	0.056
Palau	20	patch	686.167	110.032	7.373	134.388	9.565	3.001	9.620	3.001	0.001	0.001	1.207	0.112
Palau	21	patch	416.000	92.455	7.479	134.425	7.788	2.299	7.838	2.298	0.007	0.004	1.181	0.157
Palau	22	patch	587.167	149.146	7.244	134.416	18.871	4.366	18.921	4.364	0.007	0.006	1.237	0.127
Palau	23	patch	396.333	219.337	7.188	134.267	12.065	7.085	12.119	7.086	0.003	0.003	1.156	0.136
Palau	24	patch	569.667	133.816	7.255	134.314	19.275	9.981	19.330	9.982	0.002	0.002	1.269	0.065
Pohnpei	1	outer	271.167	39.306	6.918	158.345	8.013	0.935	8.072	0.935	0.001	0.001	1.036	0.011
Pohnpei	2	outer	263.000	62.360	7.001	158.147	9.832	2.425	9.814	2.396	0.077	0.170	1.114	0.080
Pohnpei	3	outer	140.833	43.462	6.784	158.127	4.146	2.533	4.182	2.516	0.023	0.029	1.104	0.180
Pohnpei	4	outer	41.833	37.712	6.842	158.350	0.851	0.509	0.889	0.516	0.022	0.014	1.046	0.055
Pohnpei	5	outer	471.667	106.402	6.980	158.120	18.065	6.078	17.715	6.247	0.409	0.620	1.055	0.034
Pohnpei	6	outer	27.667	42.231	6.828	158.332	1.092	2.225	1.113	2.235	0.039	0.082	1.120	0.015
Pohnpei	7	outer	350.000	66.906	6.988	158.329	10.692	2.512	10.751	2.512	0.000	0.001	1.029	0.008
Pohnpei	8	outer	331.000	84.150	6.771	158.229	7.853	1.609	7.896	1.609	0.017	0.012	1.394	0.203
Pohnpei	9	inner	489.333	141.706	6.927	158.108	3.105	0.926	3.161	0.926	0.003	0.004	1.469	0.189
Pohnpei	10	patch	138.000	188.583	6.976	158.160	2.171	2.789	2.219	2.792	0.011	0.012	1.113	0.104
Pohnpei	11	inner	58.333	63.990	6.808	158.161	0.567	0.455	0.617	0.460	0.009	0.009	1.420	0.142
Pohnpei	12	inner	494.833	105.433	6.859	158.133	7.659	2.200	7.715	2.200	0.003	0.003	1.590	0.244
Pohnpei	13	inner	504.500	214.712	6.954	158.282	9.652	4.797	9.709	4.793	0.002	0.005	1.360	0.305
Pohnpei	14	inner	157.167	141.841	6.848	158.340	2.655	2.794	2.711	2.795	0.003	0.004	1.336	0.207
Pohnpei	15	patch	565.667	118.822	6.894	158.106	7.455	2.078	7.506	2.078	0.008	0.004	1.171	0.089
Pohnpei	16	patch	693.667	126.840	6.973	158.128	11.874	4.361	11.926	4.364	0.007	0.004	1.460	0.131
Pohnpei	17	patch	310.000	196.544	6.940	158.085	5.469	4.271	5.467	4.190	0.061	0.124	1.150	0.063
Pohnpei	18	patch	465.333	181.924	6.818	158.153	9.467	5.378	9.521	5.378	0.005	0.006	1.511	0.160
Pohnpei	19	patch	490.500	110.132	7.013	158.239	7.133	0.863	7.178	0.864	0.015	0.011	1.302	0.096
Pohnpei	20	patch	649.000	135.036	6.989	158.317	7.695	3.800	7.742	3.801	0.012	0.006	1.466	0.197
Pohnpei	21	patch	741.667	46.945	6.976	158.244	7.369	1.748	7.413	1.749	0.016	0.016	1.290	0.094
Pohnpei	22	patch	694.833	119.854	6.978	158.133	9.355	3.302	9.411	3.301	0.004	0.003	1.243	0.185
Pohnpei	23	patch	241.333	115.303	6.864	158.354	4.757	2.411	4.773	2.451	0.043	0.057	1.231	0.103
Pohnpei	24	patch	553.167	145.912	6.933	158.330	9.131	1.950	9.180	1.957	0.010	0.015	1.279	0.228
Yap	1	outer	226.167	111.406	9.482	138.124	5.446	2.428	5.499	2.429	0.006	0.004	1.089	0.073
Yap	2	outer	515.333	52.244	9.557	138.086	11.398	0.931	11.454	0.932	0.003	0.005	1.266	0.113

Yap	3	outer	124.833	36.080	9.424	138.045	4.136	1.286	4.191	1.284	0.005	0.004	1.017	0.020
Yap	4	outer	478.000	115.211	9.446	138.039	20.077	4.826	20.131	4.828	0.005	0.004	1.258	0.119
Yap	5	outer	521.500	69.779	9.603	138.122	18.170	3.886	13.747	12.144	4.482	10.900	1.016	0.040
Yap	6	outer	335.333	68.684	9.587	138.201	17.893	7.862	17.943	7.861	0.009	0.005	1.000	0.000
Yap	7	outer	405.167	81.007	9.591	138.198	19.641	11.565	19.656	11.505	0.044	0.065	1.000	0.000
Yap	8	outer	126.000	19.980	9.454	138.090	3.513	0.715	3.552	0.723	0.020	0.012	1.064	0.010
Yap	9	outer	443.667	231.932	9.619	138.132	12.580	7.209	12.634	7.207	0.004	0.004	1.079	0.089
Yap	10	outer	435.500	90.688	9.581	138.204	13.019	3.997	13.016	3.967	0.063	0.065	1.277	0.235
Yap	11	inner	548.167	168.116	9.478	138.112	15.203	4.992	15.255	4.992	0.007	0.002	1.000	0.000
Yap	12	inner	318.667	51.821	9.657	138.160	8.954	2.521	8.817	2.481	0.196	0.260	1.019	0.045
Yap	13	inner	323.667	91.264	9.556	138.209	7.291	3.122	7.315	3.097	0.035	0.043	1.048	0.074
Yap	14	inner	652.833	184.507	9.520	138.129	2.540	0.694	2.518	0.716	0.081	0.087	1.111	0.092
Yap	15	inner	392.333	68.266	9.466	138.088	11.287	3.032	11.341	3.034	0.005	0.004	1.149	0.127
Yap	16	inner	674.667	116.330	9.579	138.185	9.522	6.071	9.573	6.073	0.008	0.007	1.457	0.140
Yap	17	inner	333.500	76.432	9.608	138.132	10.247	3.986	10.293	3.988	0.014	0.008	1.117	0.140
Yap	18	inner	333.833	96.549	9.428	138.048	8.321	2.532	8.376	2.533	0.004	0.003	1.093	0.104
Yap	19	inner	444.000	97.092	9.607	138.160	2.208	0.574	2.266	0.574	0.001	0.001	1.205	0.085
Yap	20	inner	332.333	137.769	9.618	138.133	6.646	2.424	6.696	2.419	0.009	0.007	1.000	0.000
Yap	21	inner	392.000	144.585	9.555	138.090	13.648	6.467	13.621	6.389	0.085	0.099	1.000	0.000
Yap	22	inner	291.167	85.707	9.600	138.129	9.328	5.923	9.388	5.923	0.000	0.000	1.000	0.000
Yap	23	inner	59.333	39.988	9.596	138.128	1.094	0.873	1.143	0.867	0.010	0.010	1.000	0.000
Yap	24	inner	307.833	39.179	9.588	138.181	6.003	2.498	5.927	2.585	0.135	0.225	1.171	0.046

References

Bonaldo RM, Bellwood DR (2008). Size-dependent variation in the functional role of the parrotfish *Scarus rivulatus* on the Great Barrier Reef, Australia. Marine Ecology Progress Series. 360: 237–244.

Glynn PW (1997). Bioerosion and coral-reef growth: a dynamic balance. In, C Birkeland (ed) Life and Death of Coral Reefs, Publisher, Chapman and Hall, USA. 68–95.

Hollister J, Shah T, Robitaille A, Beck M, Johnson M (2020). *elevatr: Access Elevation Data from Various APIs*. R package version 0.3.1, doi:10.5281/zenodo.4282962, https://github.com/usepa/elevatr/.

Hubbard DK (1997) Reefs as dynamic systems. In, C. Birkeland (ed) Life and Death of Coral Reefs, Publisher, Chapman and Hall, USA. 43–67.

Januchowski-Hartley FA, Graham NAJ, Wilson SK, Jennings S, Perry CT (2017) Drivers and predictions of coral reef carbonate budget trajectories. Proceedings of the Royal Society B. 284: 20162533.

Lucas CD, Python A, Redding DW (2020) Graphical outputs and spatial cross-validation for the INLA-package using INLAutils. The R Journal, preprint: arXiv:2004.02324.

Ong L, Holland KN (2010) Bioerosion of coral reefs by two Hawaiian parrotfishes: species, size differences and fishery implications. Marine Biology. 157: 1313–1323.

Perry CT, Edinger EN, Kench PS, Murphy GN, Smithers SG, Steneck RS, Mumby PJ (2012) Estimating rates of biologically driven coral reef framework production and erosion: a new census-based carbonate budget methodology and applications to the reefs of Bonaire. Coral Reefs. 31: 853–868.

R Core Team (2020) R: A language and environment for statistical computing. R Foundation for Statistical Computing, Vienna, Austria. URL https://www.R-project.org/.

van Woesik R, Cacciapaglia CW (2018) Keeping up with sea-level rise: Carbonate production rates in Palau and Yap, western Pacific Ocean. PLoS ONE 13(5): e0197077.

van Woesik R, Cacciapaglia C (2019) Carbonate production of Micronesian reefs suppressed by thermal anomalies and *Acanthaster* as sea-level rises. *PloS One* 14(11): e0224887.
